# Conjunctive changes in multiple ion channels mediate activity-dependent intrinsic plasticity in hippocampal granule cells

**DOI:** 10.1016/j.isci.2022.103922

**Published:** 2022-02-14

**Authors:** Poonam Mishra, Rishikesh Narayanan

**Affiliations:** 1 Cellular Neurophysiology Laboratory, Molecular Biophysics Unit, Indian Institute of Science, Bangalore 560012, India

**Keywords:** Biological sciences, Molecular physiology, Molecular neuroscience, Cellular neuroscience

## Abstract

Plasticity in the brain is ubiquitous. How do neurons and networks encode new information and simultaneously maintain homeostasis in the face of such ubiquitous plasticity? Here, we unveil a form of neuronal plasticity in rat hippocampal granule cells, which is mediated by conjunctive changes in HCN, inward-rectifier potassium, and persistent sodium channels induced by theta-modulated burst firing, a behaviorally relevant activity pattern. Cooperation and competition among these simultaneous changes resulted in a unique physiological signature: sub-threshold excitability and temporal summation were reduced without significant changes in action potential firing, together indicating a concurrent enhancement of supra-threshold excitability. This form of intrinsic plasticity was dependent on calcium influx through *L*-type calcium channels and inositol trisphosphate receptors. These observations demonstrate that although brain plasticity is ubiquitous, strong systemic constraints govern simultaneous plasticity in multiple components—referred here as *plasticity manifolds*—thereby providing a cellular substrate for concomitant encoding and homeostasis in engram cells.

## Highlights

• Theta-burst firing induces intrinsic plasticity in dentate gyrus granule cells
• Changes in HCN, inward-rectifier K^+^, and persistent Na^+^ channels mediate plasticity
• Ca^2+^ influx through *L*-type Ca^2+^ channels and InsP_3_ receptors governs plasticity
• Intrinsic plasticity could drive encoding and homeostasis in engram cells

## Graphical abstract

**Figure:**
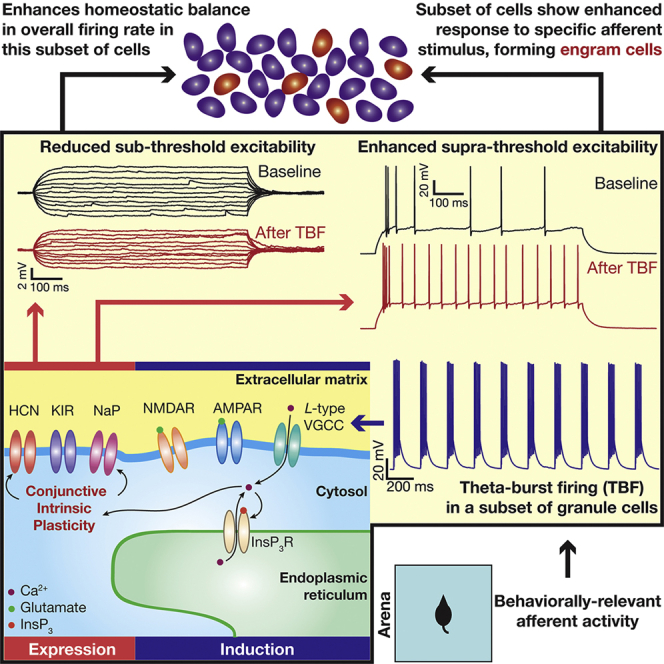

## Introduction

Granule cells in the dentate gyrus are known to be engram cell substrates for memory encoding processes (Josselyn and Tonegawa, 2020). The concomitant roles of synaptic and intrinsic forms of plasticity as putative cellular substrates of learning and memory are well established (Kim and Linden, 2007; Lisman et al., 2018; Mishra and Narayanan, 2021b; Narayanan and Johnston, 2012; Nelson and Turrigiano, 2008; Rathour and Narayanan, 2019; Titley et al., 2017; Zhang and Linden, 2003). Engram cell formation in the dentate gyrus (DG) has been shown to recruit plasticity in synaptic strength and intrinsic excitability (Josselyn and Tonegawa, 2020; Lisman et al., 2018; Titley et al., 2017). The mechanisms behind synaptic plasticity and its implications for the several distinct physiological roles of the DG have been thoroughly investigated (Aimone et al., 2014; Bliss and Lomo, 1973; Schmidt-Hieber et al., 2004; Tonegawa et al., 2018). In contrast, despite the well-recognized role of intrinsic plasticity in engram cell formation, the cellular correlates (ion channel subtypes, for instance) underlying activity-dependent plasticity of intrinsic excitability in the DG remains unknown.

Here, seeking to fill this lacuna, we focused on identifying behaviorally relevant activity patterns that could induce intrinsic plasticity in DG granule cells and on mechanistically understanding such plasticity. In search of behaviorally relevant activity patterns, we first noted that theta frequency (4–10 Hz) oscillations are widely prevalent in the DG (Bland, 1986; Buzsaki, 2002; Colgin, 2013, 2016; Sainsbury and Bland, 1981; Winson, 1974, 1978) and granule cells exhibit theta-modulated bursts under *in vivo* conditions (Diamantaki et al., 2016; Pernia-Andrade and Jonas, 2014; Zhang et al., 2020). Although the impact of theta patterned stimuli has been widely studied with reference to synaptic plasticity (Beck et al., 2000; Davis et al., 2004; Greenstein et al., 1988; Larson and Munkacsy, 2015; McHugh et al., 2007; Pavlides et al., 1988; Shors and Dryver, 1994), the impact of theta burst firing (TBF) in eliciting plasticity in neuronal intrinsic properties has not been explored. Therefore, employing whole-cell patch-clamp recordings, we explored the impact of intracellularly initiated theta-modulated burst firing, in the absence of synaptic stimulation, on intrinsic neuronal properties of DG granule cells.

We found that TBF reliably induced intrinsic plasticity in DG granule cells, manifesting as a significant reduction in sub-threshold excitability and temporal summation, and a surprising absence of significant reductions in action potential firing. Together these pointed to independent and contrasting changes in sub- and supra-threshold excitability, with a reduction in sub-threshold excitability nullified by independent increases in supra-threshold excitability. We provide strong lines of evidence, based on systematic analyses of signature electrophysiological characteristics and results of experiments involving pharmacological agents, which supports conjunctive changes in HCN, inward-rectifier potassium, and persistent sodium channels mediating this form of plasticity. Finally, we demonstrated that TBF-induced intrinsic plasticity was dependent on the influx of cytosolic calcium, with *L-*type calcium channels and intracellular calcium stores contributing to calcium influx. Our results unveil the expression of conjunctive plasticity in multiple ion channels, responding to the *same* activity pattern, thereby establishing a *plasticity manifold* involving strong rules governing concomitant plasticity in different components (Mishra and Narayanan, 2021b). Such a plasticity manifold ensures that neural components have specific rules associated with how the expression of plasticity in these components is co-regulated, thereby providing a molecular substrate for neurons to traverse across different stable states (say, each *encoding* different contexts without altering activity *homeostasis*) within the neuronal intrinsic manifold (Jazayeri and Afraz, 2017; Krakauer et al., 2017; Mishra and Narayanan, 2021b; Rathour and Narayanan, 2019). We postulate that the activity-dependent increase in supra-threshold excitability could act as a putative substrate for the emergence of engram cells, and the associated reduction in sub-threshold excitability could concomitantly provide homeostatic balance to the encoding process.

## Results

**Figure 1:**
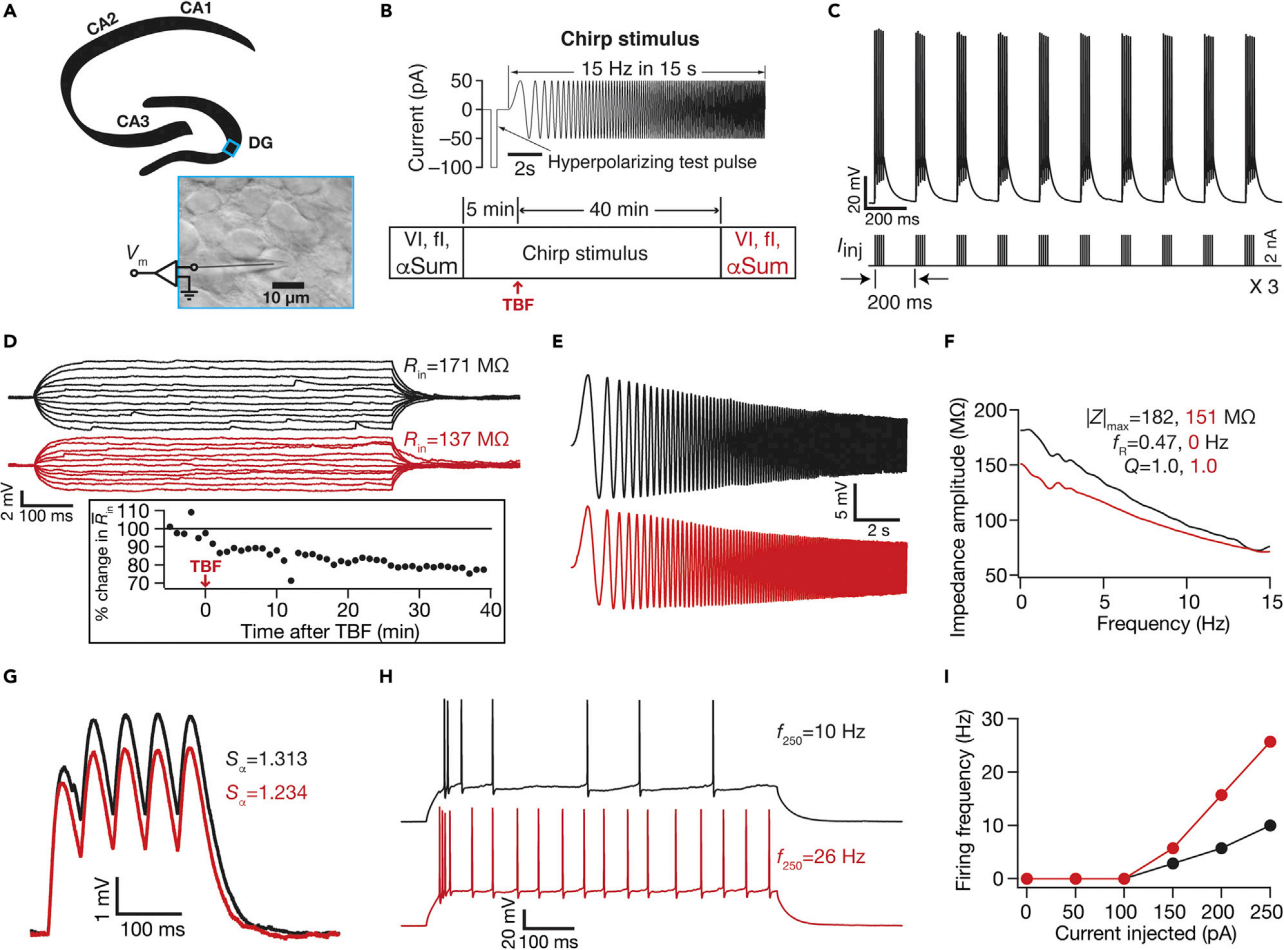
Illustration of TBF inducing contrasting changes in sub- vs. supra-threshold excitability in a DG granule cell (A) Illustration showing whole-cell patch clamp recording from a DG granule cell (B) Top: the chirp stimulus, including a large hyperpolarizing pulse for measuring ${\bar{R}}_{\text{in}}$. Bottom: experimental protocol showing measurements and time course of TBF experiments (C) Granule-cell voltage response (top) to theta-patterned current injection (bottom), showing 50 action potentials generated during TBF (D) Granule-cell voltage responses to 700-ms current pulses of amplitude varying from −25 to +25 pA (in steps of 5 pA), before (black) and 40 min after (red) TBF. Inset: temporal evolution of ${\bar{R}}_{\text{in}}$ during the experiment (E) Granule-cell voltage response to the chirp current before (black: 0–5 min average) and 40 min after (red: 40–45 min average) TBF (F) Impedance amplitude computed from the current stimulus shown in panel B (top) and the voltage responses shown in panel (E) (G) Granule-cell voltage responses to five alpha-current injections arriving at 20 Hz, recorded before (black) and 40 min after (red) TBF (H) Granule-cell voltage responses to a 700-ms current pulse of 250 pA, before (black) and 40 min after (red) TBF (I) Frequency of AP firing plotted as a function of injected current amplitude for the example cell. All traces and measurements shown in this figure are from a single experiment

We performed whole-cell current clamp recordings from the somata of DG granule cells. The behaviorally relevant activity pattern that we explored in our analyses is theta-burst firing (TBF), initiated by theta-patterned supra-threshold current injections (Figure 1C, bottom) to induce action potential (AP) bursts (Figure 1C, top). We first asked if the intrinsic properties of DG granule cells change in response to these behaviorally relevant activity patterns.

### TBF elicited contrasting patterns of plasticity in sub- vs. supra-threshold physiological properties of granule cells

**Figure 2:**
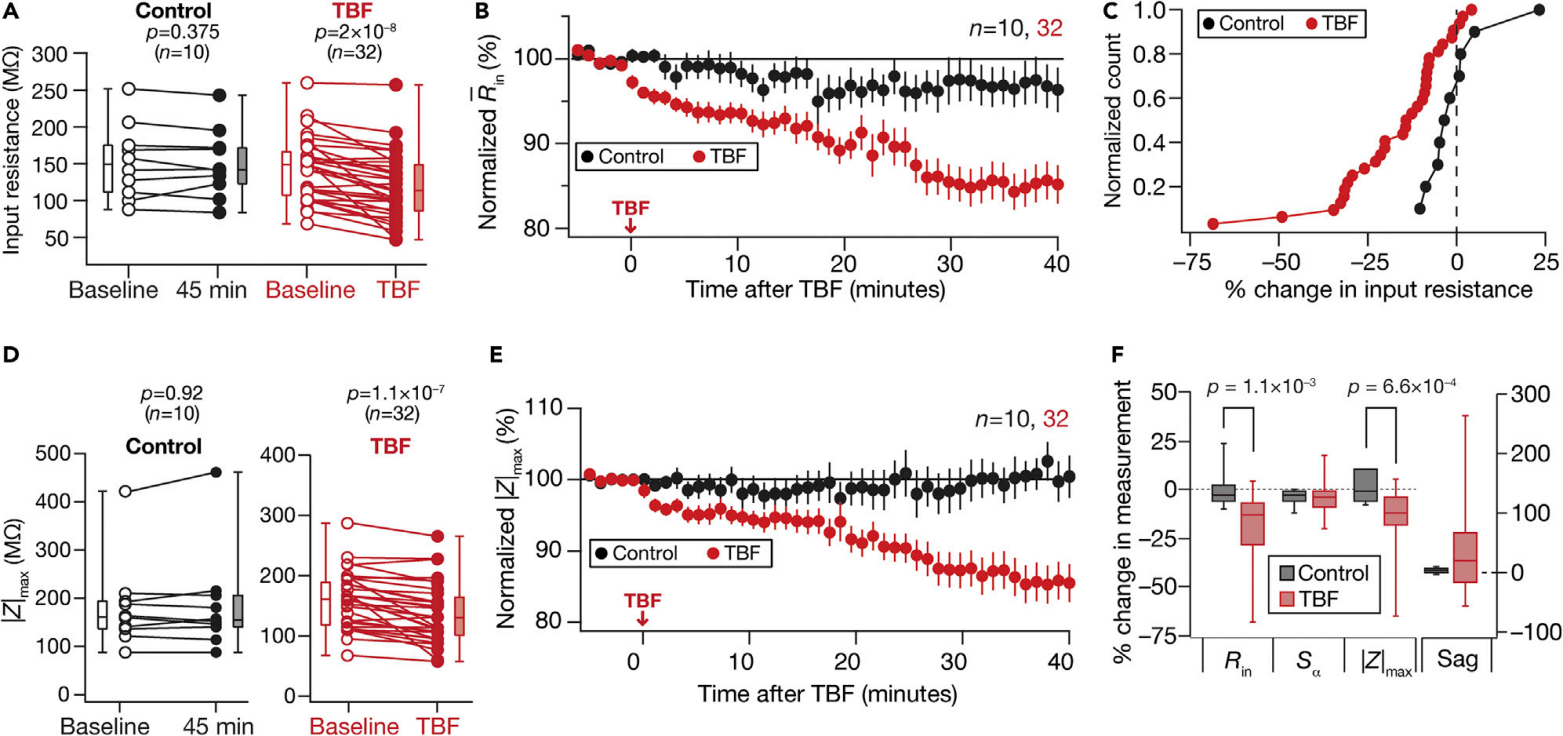
TBF reduced sub-threshold excitability in DG granule cells For all panels, the Control (black) group (*n =* 10) corresponds to experiments where no protocol was applied through the 45-min period of the experiment, and TBF (red) group (*n =* 32) is for neurons subjected to TBF. For the TBF group, “Baseline” measurements were obtained before TBF and “TBF” measurements were after TBF (A) Population data representing change in *R*_in_ at the beginning (empty circles) and the end (filled circles) of the experiment. Two-way mixed ANOVA, interaction p = 0.0097 (B) Temporal evolution of percentage change in ${\bar{R}}_{in}$ (mean ± SEM) (C) Normalized count of neurons from panel A plotted as functions of percentage change in *R*_in_ both for the Control and TBF cases (D and E) Same as (A–B), representing |*Z*|_max_ measurements. Two-way mixed ANOVA, interaction p = 0.0032 (F) Plots comparing percentage change in various sub-threshold measurements from their initial values to end of experiment values, for both Control and TBF experiments. The list of symbols and corresponding measurements is enumerated in Table S1. The Wilcoxon signed rank test was used for *p*-value calculation in panels A and D, for comparing measurements from the same set of cells. The Wilcoxon rank-sum test was employed for *p*-value calculation in panel F, to compare percentage changes in the Control vs. TBF group. Details of statistics associated with these measurements are provided in Table S1

We found a significant long-term reduction in input resistance (*R*_in_) of granule cells post-TBF that persisted for more than 40 min (Figures 1D and 2A–2C). The resting membrane potential (RMP) also depolarized (∼7 mV) through this period, but all measurements were obtained at the initial RMP to avoid confounds because of altered driving forces to different ion channels (Ashhad et al., 2015; Brager et al., 2012; Clemens and Johnston, 2014; Fan et al., 2005; Malik and Johnston, 2017; Mishra and Narayanan, 2015; Narayanan et al., 2010; Narayanan and Johnston, 2007, 2008). Consistent with the reduction in *R*_in_, we also found a concomitant persistent reduction in impedance amplitude (Figures 1E*–*1F), which was quantified using maximal impedance amplitude (|*Z*|_max_; Figures 2D and 2E), and in temporal summation (*S*_α_; Figures 1G and 2F) measured from the neuron’s response to five alpha excitatory postsynaptic currents.

**Figure 3:**
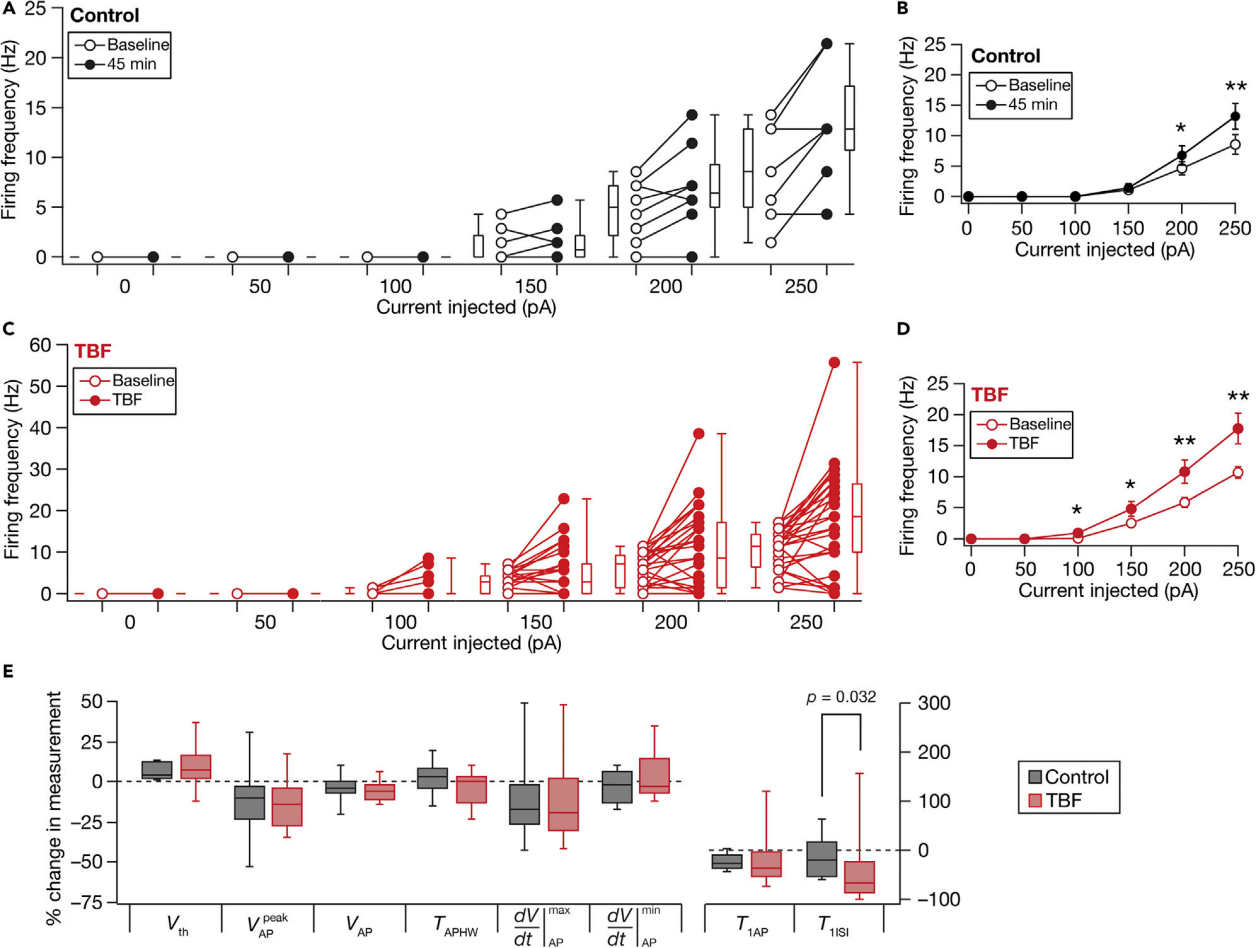
Impact of TBF on supra-threshold excitability in DG granule cells For all panels, the Control (black) group (*n =* 10) corresponds to experiments where no protocol was applied through the 45-min period of the experiment, and TBF (red) group (*n =* 32) is for neurons subjected to TBF (same population of cells from Figure 2). For the TBF group, “Baseline” measurements were obtained before TBF and “TBF” measurements were after TBF (A) Population data representing changes in the action potential firing frequency at the beginning (empty circles) and end (filled circles) of the experiment, for six values of current injection (B) Summary statistics (mean ± SEM) of action potential firing frequency plotted as a function of injected current amplitude for the Control group. *∗*p < 0.05; ∗∗p <0.005. Student’s *t* test (C and D) Same as (A–B) for the TBF group where the cells were subjected to TBF. For each of the five (50, 100, 150, 200, and 250 pA) current injections, there was no significant interaction between time (0 vs. 45 min) and protocol (Control vs. TBF) factors when assessed with two-way mixed ANOVA (E) Plots comparing percentage change in various supra-threshold measurements from their initial values to end of experiment values, for both Control and TBF experiments. The list of symbols and corresponding measurements is enumerated in Table S1. The Wilcoxon rank-sum test was employed for *p*-value calculation in panel E, to compare percentage changes in the Control vs. TBF group. Details of statistics associated with these measurements are provided in Table S2

**Figure 4:**
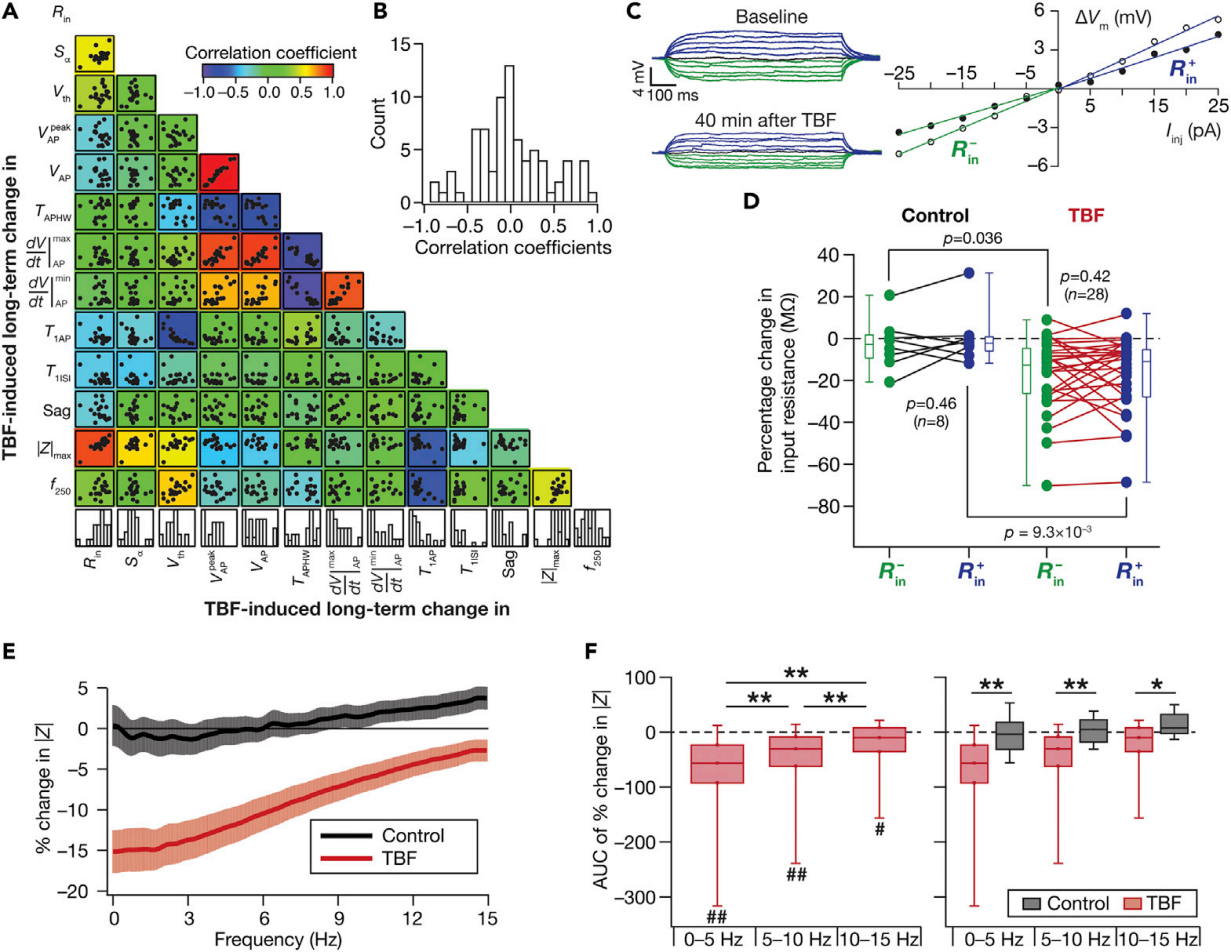
Signature changes in several physiological measurements point to TBF-induced changes in multiple ion channels, with plasticity in resting conductances as a candidate mechanism behind the reduction in sub-threshold excitability (A and B) Differential pair-wise correlations among TBF-induced changes in 13 electrophysiological measurements. (A) Pairwise scatterplot matrices of TBF-induced changes in 13 sub- and supra-threshold measurements recorded from granule cells (*n =* 20). These scatterplot matrices are overlaid on the corresponding color-coded correlation matrices. The bottom most row presents the histogram of percentage changes for corresponding measurements spanning their respective ranges. Symbols: input resistance, *R*_in_; summation ratio of αEPSPs, *S*_α_; AP threshold, *V*_th_; AP peak, $V_{AP}^{\text{peak}}$; AP amplitude, *V*_AP_; AP half-width, *T*_APHW_; peak dV/dt, ${\frac{\mathrm{d}V}{\mathrm{d}t}|}_{AP}^{\max}$; min dV/dt, ${\frac{\mathrm{d}V}{\mathrm{d}t}|}_{AP}^{\min}$; latency to first spike, *T*_1AP_; first ISI, *T*_1ISI_; percentage sag, Sag; Maximal impedance amplitude, |*Z*|_max_; firing frequency for a 250-pA current injection, *f*_250_. (B) Distribution of correlation coefficient values for 13 measurements corresponding to the pairwise scatterplots shown in panel (A). (C**–**F) Analyses of changes in steady-state and frequency-dependent measurements point to HCN channels as a candidate mechanism behind TBF-induced reduction in sub-threshold excitability. (C) Voltage responses of an example neuron to 700 ms current pulses of amplitude varying from −25 pA to +25 pA (in steps of 5 pA), recorded before (*Baseline*) and 40 min after TBF. The responses colored blue and green are for positive and negative current injections, respectively. Input resistance values, computed from depolarizing $\left(R_{in}^{+}\right)$ and hyperpolarizing $\left(R_{in}^{-}\right)$ responses, were slopes of the plots depicting steady-state voltage responses as functions of positive and negative current injections, respectively (bottom). $R_{in}^{+}\;\text{and}\;R_{in}^{-}$ were calculated for traces obtained before (open circles) and after (closed circles) TBF (bottom). (D) Population level analysis to quantify percentage changes (at the end of experiment, compared to the measurement at the beginning) in $R_{in}^{+}$ (blue) and $R_{in}^{-}$ (green) independently for Control (*n =* 8) and TBF (*n =* 28) experiments. The *p*-values presented for comparisons within each group (either Control or TBF) correspond to Wilcoxon signed rank test and *p*-values across groups are for Wilcoxon rank-sum test. Two-way mixed ANOVA, interaction p = 0.563. (E) Percentage changes in impedance amplitude as a function of frequency for Control (no protocol) and TBF groups. In the Control group, the percentage changes were computed as the change in impedance amplitude measured at 45 min, compared to the initial measurements. In the TBF group, the percentage changes are between those measured before and 40 min after TBF. (F) Quantification of percentage changes in |*Z*| within three frequency bands (0–5, 5–10, and 10–15 Hz). The value for each neuron were computed as the AUC of percentage change in |*Z*|. Left: plot showing the quartiles of AUC of percentage change in |*Z*| for neurons in the TBF group. The symbol “#” refers to the outcomes of Wilcoxon rank-sum test on whether TBF-induced changes in |*Z*| within that frequency band were significantly different from zero. #p <0.05; ##p <0.001. The symbol “∗” refers to the outcomes of Wilcoxon signed rank test, assessing whether TBF-induced changes in |*Z*| across the different frequency bands were significantly different from one another. ∗∗p <0.001. Right: plot showing the quartiles of AUC of percentage change in |*Z*| for neurons in the Control and TBF groups. The symbol “∗” refers to the outcomes of Wilcoxon rank-sum test, assessing whether TBF-induced changes in |*Z*| within each frequency band were significantly different between the Control and TBF groups. ∗p <0.05; ∗∗p <0.005. For the Control group, changes in |*Z*| within none of the three frequency bands were significantly different from zero, or significantly different from one another. The representation has been split into two separate graphs to avoid clutter of symbols denoting statistical test outcomes

Despite these significant reductions in sub-threshold excitability measurements (Figure 2 and Table S1), which should have typically resulted in reduced AP firing (Fan et al., 2005; Narayanan and Johnston, 2007), we found a surprising absence of significant reductions in action potential firing rates after TBF compared to a time-matched Control group (Figures 1H, 1I, 3 and Table S2). This was coupled with changes in the firing patterns of neurons, whereby there was an occurrence of doublets at the beginning of the voltage response, quantified as a significant reduction in the first ISI after TBF (Figure 3E). This form of contrasting plasticity was consistently reproducible (n = 32; Tables S1 and S2) with measurements significantly different from 45-min control recordings (*n* = 10) where no plasticity induction protocol was employed (Figure 2 and 3). There was pronounced cell-to-cell variability in the amount of plasticity expressed in each of the different measurements we employed, pointing to the expression of *plasticity heterogeneities* in the granule cell population (Figures 2, 3, and 4). However, heterogeneities in TBF-induced sub- or supra-threshold plasticity did not show strong correlations with heterogeneities in baseline neuronal intrinsic properties (Figure S1). In one set of experiments, we performed physiological measurements before and after TBF at the respective RMP values *without* injecting current to maintain membrane potential at the initial value of the RMP (Figure S2). We found consistent TBF-induced reductions in *R*_in_ and concomitant increases in firing rate, even when measurements were made at respective RMP values (Figure S2). Together, our results show that TBF yielded concomitant yet contrasting plasticity of sub- and supra-threshold intrinsic excitability in DG granule cells.

### TBF-induced plasticity in AP firing rate is consequent to a competition between changes in sub- and supra-threshold excitability

We quantified changes in 13 different sub- and supra-threshold measurements to assess the multifarious TBF-induced changes in granule cell physiology. Pairwise scatterplots of TBF-induced concomitant changes in these measurements revealed pronounced cell-to-cell variability in the expression of plasticity in each of these measurements (Figures 2 and 3). Are heterogeneities in TBF-induced plasticity in these measurements related to each other, whereby specific pairs of measurements exhibited correlated changes despite each measurement manifesting heterogeneities? To assess this, we computed pair-wise Pearson’s correlation between the TBF-induced percentage changes in each of the 13 individual measurements (Figures 4A and 4B). Among measurements that exhibited strong positive correlations in their TBF-induced changes were between measures of sub-threshold excitability (*R*_in_, |*Z*|_max_, *S*_α_) and between AP measurements $\left(V_{AP},V_{AP}^{\text{peak}},V_{AP},{\frac{\mathrm{d}V}{\mathrm{d}t}|}_{AP}^{\max},{\frac{\mathrm{d}V}{\mathrm{d}t}|}_{AP}^{\min}\right)$. On the other hand, TBF-induced changes in AP half-width showed strong negative correlations with each of changes in *V*_AP_, $V_{AP}^{\text{peak}}$, *V*_AP_, ${\frac{\mathrm{d}V}{\mathrm{d}t}|}_{AP}^{\max}$, ${\frac{\mathrm{d}V}{\mathrm{d}t}|}_{AP}^{\min}$, and changes in latency to the first spike exhibited strong negative correlations with changes in AP threshold, and AP firing. Importantly, we noted strong correlations between changes in AP firing rate and changes in AP threshold, suggesting that changes in AP threshold could be mediating the enhanced firing rate despite a reduction in sub-threshold excitability. The two groups of strongly correlated changes (Group 1: sub-threshold measurements; Group 2: AP measurements), in conjunction with this strong relationship between AP firing and AP threshold changes, suggested that TBF-induced changes in the firing rate were regulated by two concurrent yet competing mechanisms. The first is a reduction in sub-threshold excitability that reduces firing rate and the second is an independent increase in supra-threshold excitability. We noted that the surprising absence of strong correlations (positive or negative) between changes in sub- vs. supra-threshold measurements could be consequent to this competition. Together, the contrasting changes introduced in sub- vs. supra-threshold measurements, with potential competition between different mechanisms that drove sub-vs. supra-threshold excitability, pointed to conjunctive changes in multiple ion channels that mediated TBF-induced intrinsic plasticity.

### Signature changes in several physiological measurements point to changes in resting conductances as a candidate mechanism behind TBF-induced reduction in sub-threshold excitability

Our results on reduction in sub-threshold excitability point to changes in a conductance that is active at rest. If this conductance were depolarization-activated, TBF-induced changes in voltage responses to depolarizing current pulses (where the channel is activated/deactivated) would be larger compared to responses to hyperpolarizing pulses (which are beyond the channel’s activation/deactivation range). On the other hand, a hyperpolarization-activated conductance would show an opposite response profile, whereby there would be larger changes to hyperpolarized current injections compared to depolarized current injections. If the magnitude of changes were invariant to hyperpolarizing and depolarizing pulses, it would point to changes in a voltage-independent conductance or in a conductance whose activation/deactivation profile is linear within the range of measurement. To assess this, we calculated TBF-induced changes in two measures of input resistance, computed independently from voltage responses to depolarizing $\left(R_{in}^{+}\right)$ or hyperpolarizing $\left(R_{in}^{-}\right)$ current injections (Figure 4C). We performed this analysis both for Control and TBF experiments and observed no significant difference between percentage changes in $R_{in}^{+}\;vs.\;R_{in}^{-}$ within either control or TBF experiments (Figures 4C and 4D). However, consistent with prior observations on *R*_in_ (Figure 2), the percentage changes in $R_{in}^{+}\;or\;R_{in}^{-}$ across control vs. TBF experiments were significantly different (Figure 4D). These observations point to TBF-induced changes in a resting conductance whose activation profile is broadly linear in the range explored here, also ruling out the involvement of most depolarization-activated conductances.

Which resting conductance could be contributing to TBF-induced reduction in sub-threshold excitability? Focusing on TBF-induced changes in sub-threshold measurements, we observed a depolarizing shift in the RMP and reductions in *R*_in_, |*Z*|_max_, and *S*_α_. These changes are broadly consistent with an increased current passage through HCN channels, which are hyperpolarization-activated channels that mediate an inward current that is active under resting conditions. Specifically, it has been shown that increases in HCN channels and the associated *h* current result in depolarization of RMP, a reduction of *R*_in_, |*Z*|_max_ and *S*_α_ (Magee, 1998; Narayanan and Johnston, 2007; Mishra and Narayanan, 2021a; Stegen et al., 2012). We asked if other TBF-induced sub-threshold changes were also consistent with changes in HCN channels. Specifically, if TBF had altered HCN channels, which mediate a slow conductance, TBF-induced changes in impedance amplitude would be predominantly in the lower frequencies (Narayanan and Johnston, 2008). Therefore, we quantified changes in impedance amplitude as a function of frequency. We observed significantly higher TBF-induced changes in impedance amplitude at lower frequencies compared to changes at higher frequencies (Figures 4E and 4F), pointing to changes in HCN channels as a putative substrate for TBF-induced sub-threshold changes. In contrast, changes in impedance amplitude were negligible in control experiments where no activity-dependent protocol was presented (Figures 4E and 4F).

Together, signature TBF-induced changes in several physiological measurements (Figures 2, 3, and 4; Table S1) strongly point to a role for changes in HCN channels and other resting conductances as mechanisms underlying the observed reduction in sub-threshold excitability.

### Synergistic interactions between HCN and inward-rectifier potassium channels mediate TBF-induced reduction in sub-threshold excitability of DG granule cells

**Figure 5:**
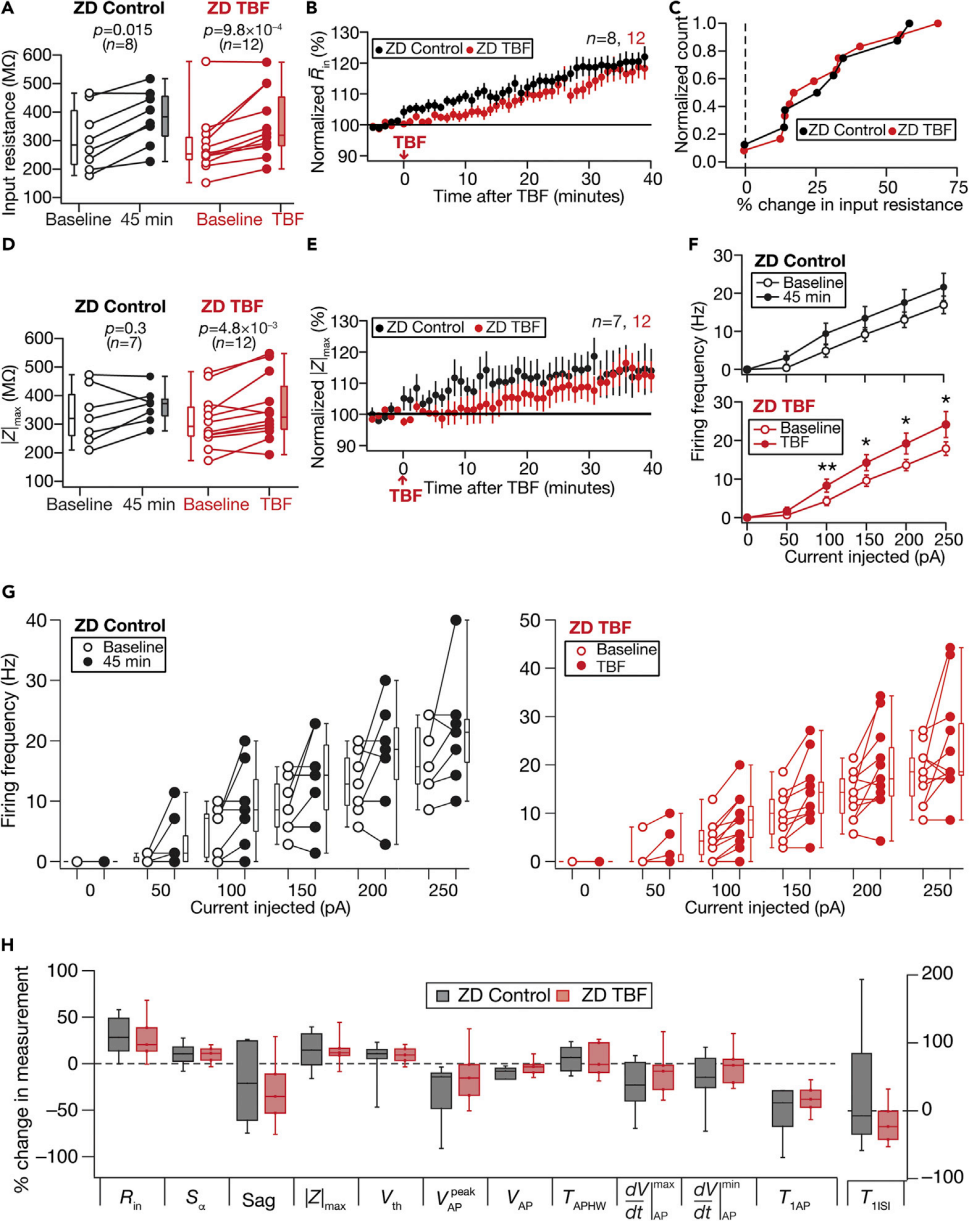
Activity-dependent reduction in sub-threshold excitability was blocked in the presence of ZD7288 Control and TBF group experiments were performed in the presence of 20-μM ZD7288 in the bath and pipette solution. The Control groups (black) correspond to experiments where no protocol was applied through the 45-min period of the experiment, and TBF Group (red) is for neurons subjected to TBF, with all experiments performed in the presence of ZD7288 (A) Population data representing change in *R*_in_ at the beginning (empty circles) and end (filled circles) of the experiment. Two-way mixed ANOVA, interaction p = 0.9183 (B) Temporal evolution of percentage changes in ${\bar{R}}_{\text{in}}$ (mean ± SEM) (C) Normalized count of neurons from panel A plotted as functions of percentage change in *R*_in_ (D and E) Same as panels A–B, representing |*Z*|_max_ measurements. Two-way mixed ANOVA, interaction p = 0.664 (F) Summary statistics (mean ± SEM) of action potential firing frequency plotted as a function of injected current amplitude for both groups. ∗p <0.05; ∗∗p <0.005. Student’s *t* test (G) Population data representing changes in the action potential firing frequency at the beginning (empty circles) and the end (filled circles) of the experiment, for six values of current injection, for the Control (left) and the TBF (right) groups. For each of the five (50, 100, 150, 200, and 250 pA) current injections, there was no significant interaction between time (0 vs. 45 min) and protocol (Control vs. TBF) factors when assessed with two-way mixed ANOVA. (H) Plots comparing percentage change in various measurements from their baseline-to-final values are provided for each measurement. The Wilcoxon signed rank test was used for *p*-value calculation in panels A and D, for comparing measurements from the same set of cells. The Wilcoxon rank-sum test was employed for *p*-value calculation in panel H, to compare percentage changes in the Control vs. TBF group

ZD7288, an HCN-channel blocker, significantly reduces sub- and supra-threshold excitability of DG granule cells (Mishra and Narayanan, 2021a). Encouraged by these strong physiological lines of evidence on the potential role of resting conductances in sub-threshold plasticity (Figure 4C–4F), we first tested the time-dependent effect of ZD7288 on the intrinsic properties of DG granule cells. In these control experiments, we found a slow yet significant increase in *R*_in_ over a period of 45 min (Figures 5A–5C), which also reflected as increases in |*Z*|_max_ (Figure 5D and 5E). Next, we performed TBF experiments in the presence of ZD7288, and found *R*_in_ and |*Z*|_max_ to increase with time, with the strength of these increases across TBF experiments statistically insignificant when compared to control experiments performed in the presence of ZD7288 (Figures 5A–5E and 5H). These results demonstrated that TBF-induced reduction in sub-threshold excitability (Figure 2) did not express in the presence of ZD7288. Although there was a significant TBF-induced enhancement in supra-threshold excitability (Figure 5F), there was no interaction between the time-matched “ZD control” and “ZD TBF” groups (Figures 5F and 5G).

**Figure 6:**
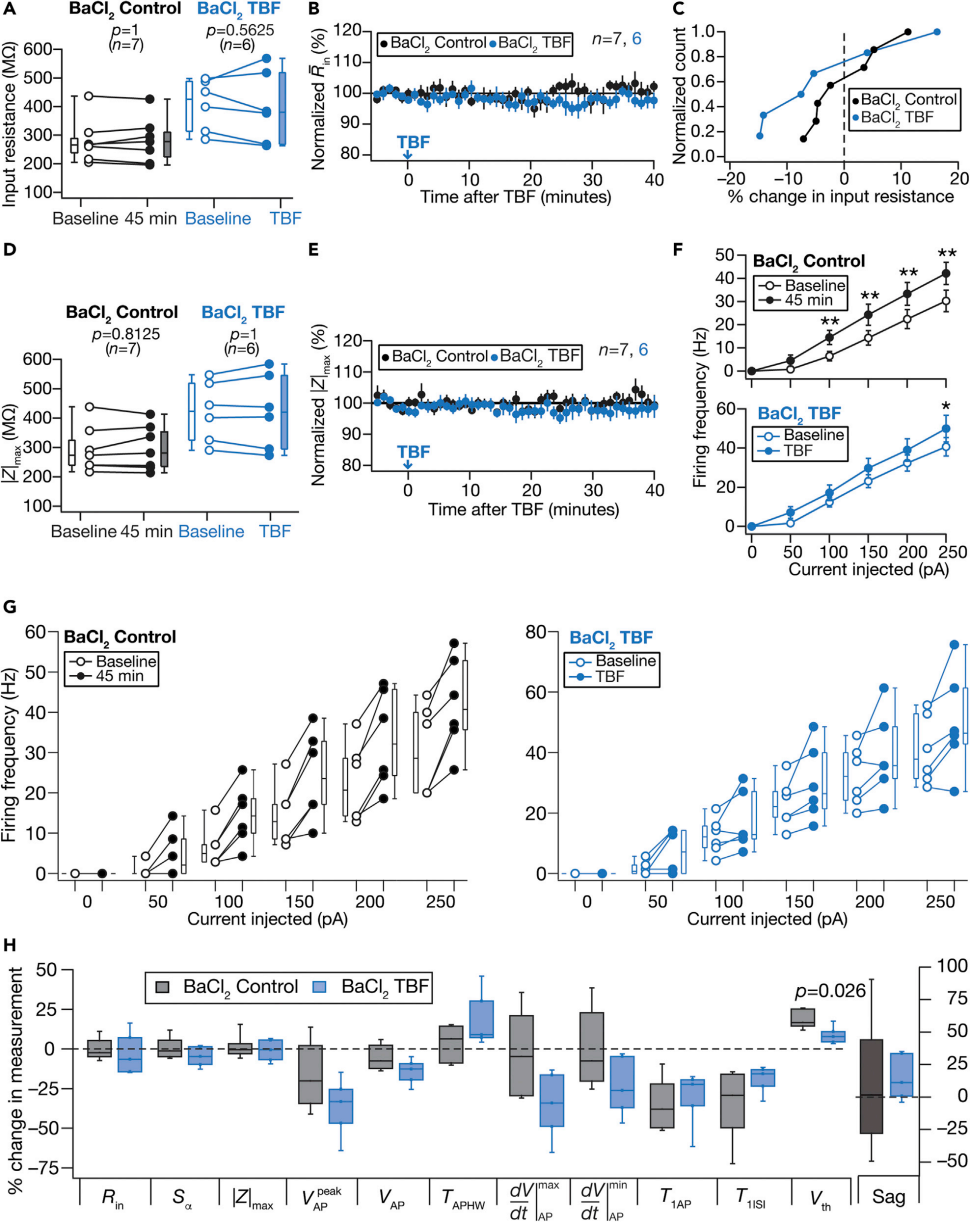
Activity-dependent reduction in sub-threshold excitability was blocked in the presence of barium chloride (BaCl_2_) Control and TBF group experiments were performed in the presence of 50-μM BaCl_2_ (blue) in the bath. The Control groups (black) correspond to experiments where no protocol was applied through the 45-min period of the experiment, and TBF group is for neurons subjected to TBF, with all experiments performed in the presence of BaCl_2_ (A) Population data representing change in *R*_in_ at the beginning (empty circles) and end (filled circles) of the experiment. Two-way mixed ANOVA, interaction p = 0.637 (B) Temporal evolution of percentage changes in ${\bar{R}}_{in}$ (mean ± SEM) (C) Normalized count of neurons from panel A plotted as functions of percentage change in *R*_in_ (D and E) Same as panels A–B, representing |*Z*|_max_ measurements. Two-way mixed ANOVA, interaction p = 0.879 (F) Summary statistics (mean ± SEM) of action potential firing frequency plotted as a function of injected current amplitude for both groups. ∗p <0.05; ∗∗p <0.005. Student’s *t* test (G) Population data representing changes in the action potential firing frequency at the beginning (empty circles) and the end (filled circles) of the experiment, for six values of current injection, for the Control (left) and the TBF (right) groups. For each of the five (50, 100, 150, 200, and 250 pA) current injections, there was no significant interaction between time (0 vs. 45 min) and protocol (Control vs. TBF) factors when assessed with two-way mixed ANOVA (H) Plots comparing percentage change in various measurements from their baseline-to-final values are provided for each measurement. The Wilcoxon signed rank test was used for *p*-value calculation in panels A and D, for comparing measurements from the same set of cells. The Wilcoxon rank-sum test was employed for *p*-value calculation in panel H, to compare percentage changes in the Control vs. TBF group.

As inward rectifier potassium (K_ir_) channels, which mediate another resting conductance, are known regulators of granule cell excitability (Mishra and Narayanan, 2021a; Stegen et al., 2009, 2012; Young et al., 2009), we pharmacologically assessed their role in TBF-induced plasticity using 50-μM BaCl_2_, a blocker of K_ir_ channels. With long-term control experiments performed in the presence of BaCl_2_, although there were no significant changes in sub-threshold measurements over the 45 min period (Figures 6A–6E), there were significant increases in firing rate over the period (Figure 6F; BaCl_2_ Control). With TBF experiments, we found that the presence of BaCl_2_ blocked TBF-induced reduction in *R*_in_ and other sub-threshold measurements (Figure 6). There was no interaction between the time-matched “BaCl_2_ control” and “BaCl_2_ TBF” groups for any of the different current injections (Figures 6F and 6G). These experiments demonstrated that barium-sensitive channels play a critical role in mediating TBF-induced plasticity of intrinsic properties.

Together, our physiological analyses (Figure 4) and TBF experiments in the presence of pharmacological blockers of HCN or K_ir_ channels (Figures 5 and 6) provided lines of evidence for a critical role for synergistic interactions between these two resting conductances in mediating TBF-induced changes in sub-threshold excitability. Whereas TBF-induced changes in sub-threshold excitability could be explained either by increases in HCN or K_ir_ channels, TBF-induced *depolarization* in RMP could be explained by an increase in the inward HCN, but not the outward K_ir_ channels. Therefore, it is possible that while changes in both HCN and K_ir_ channels synergistically contribute to the reduction in sub-threshold excitability, relative dominance of changes to HCN channels results in TBF-induced depolarization of RMP.

### Plasticity in NaP channels results in TBF-induced increase in supra-threshold excitability of DG granule cells

**Figure 7:**
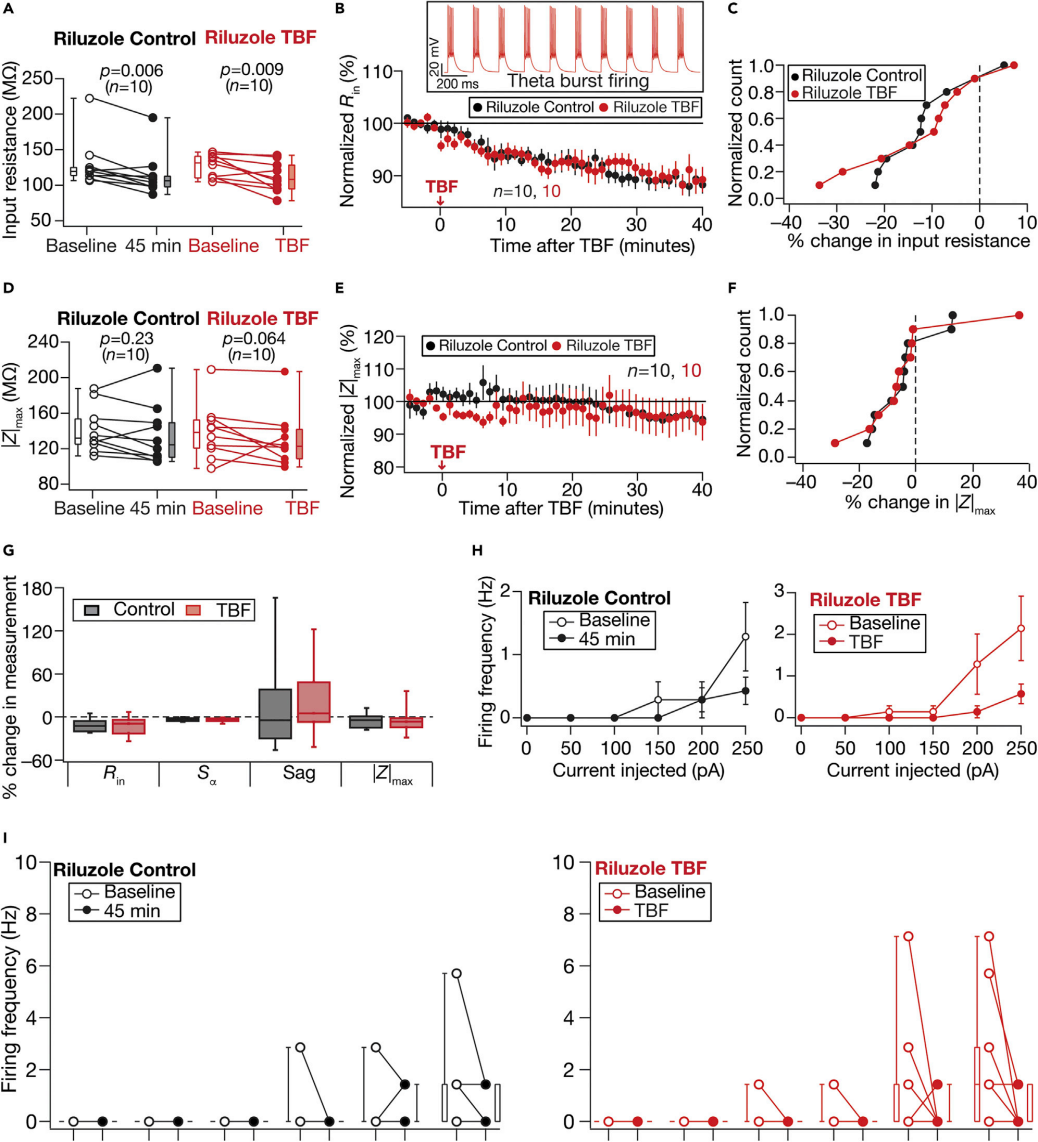
Activity-dependent intrinsic plasticity was blocked by Riluzole, a persistent sodium channel blocker For all panels, the Riluzole Control (black) group (*n =* 10) corresponds to experiments where no protocol was applied through the 45-min period of the experiment, and Riluzole TBF (red) group (*n =* 10) is for neurons subjected to TBF. All experiments were performed in the presence of 20-μM Riluzole in the bath (A) Population data representing change in *R*_in_ at the beginning (empty circles) and end (filled circles) of the experiment. Two-way mixed ANOVA, interaction p = 0.966 (B) Temporal evolution of percentage change in ${\bar{R}}_{in}$ (mean ± SEM). Inset: Membrane voltage recorded from a granule cell in response to theta-patterned current injection in the presence of riluzole in the ACSF, showing 50 action potential elicited during a theta burst firing (TBF) pattern. Similar to other cases, this train was repeated thrice (total 150 action potentials) with 10 s inter-train intervals to form the TBF protocol. The presence of riluzole did not affect the ability of granule cells to generate theta burst firing (C) Normalized count of neurons from panel A plotted as a function of percentage change in *R*_in_ (D–F) Same as (A–C) representing |*Z*|_max_ measurements. Two-way mixed ANOVA, interaction p = 0.79 (G) Plots comparing percentage change in various sub-threshold measurements from their initial values to the end of experiment values (H) Summary statistics (mean ± SEM) of action potential firing frequency plotted as a function of injected current amplitude for both groups. ∗p <0.05; ∗∗p <0.005. Student’s *t* test (I) Population data representing changes in the action potential firing frequency at the beginning (empty circles) and end (filled circles) of the experiment, for six values of current injection, for the Control (left) and TBF (right) groups. For each of the five (50, 100, 150, 200, and 250 pA) current injections, there was no significant interaction between time (0 vs. 45 min) and protocol (Control vs. TBF) factors when assessed with two-way mixed ANOVA. The Wilcoxon signed rank test was used for *p*-value calculation in panels A and D, for comparing measurements from the same set of cells. The Wilcoxon rank-sum test was employed for *p*-value calculation in panel G, to compare percentage changes in the Control vs. TBF group

Our observations on TBF-induced enhancement in supra-threshold excitability pointed to changes in a regenerative conductance. Motivated by the expression of a persistent sodium (NaP) channel in DG granule cells predominantly in the axonal initial segment, and the role of these channels in regulating AP firing properties (Artinian et al., 2011; Crill, 1996; Ellerkmann et al., 2003; Epsztein et al., 2010; Kress et al., 2010; Mishra and Narayanan, 2021a), we assessed TBF-induced plasticity in the presence of riluzole, a blocker of NaP channels (Song et al., 1997; Urbani and Belluzzi, 2000). We observed a significant reduction in *R*_in_ (Figures 7A–7C; Riluzole Control), but not |*Z*|_max_ (Figures 7D–7F; Riluzole Control) over a 45-min period long-term control, even when we pre-treated the slice with riluzole before beginning our recordings. We performed TBF experiments in the presence of riluzole and compared these outcomes with riluzole control experiments (Figure 7). We confirmed that the TBF protocol elicited 150 APs through the protocol period, and there was no difference in TBF firing pattern in the presence of riluzole (Figure 7B). Comparing Control vs. TBF experiments, we found that TBF-induced reductions in *R*_in_ (Figures 7B and BC) and |*Z*|_max_ (Figures 7D–7F) were abolished in the presence of riluzole. More importantly, the firing rate changes induced by TBF (Figure 3) were completely abolished in the presence of riluzole with no further increase in the firing rate post-TBF (Figure 7H–I). We found that riluzole abolished TBF-induced changes in all sub-threshold measurements (Figure 7G), as no significant difference was observed between Control and TBF groups in the presence of riluzole. We could not quantify changes in AP properties as neurons did not fire at 250 pA for most of the recordings (both Control and TBF groups).

Together, the direction of changes in several physiological measurements (Figures 2, 3, 4, Tables S1 and S2), TBF experiments with ZD7288 (Figure 5), BaCl_2_ (Figure 6), or riluzole (Figure 7) provided lines of evidence for conjunctive changes in HCN, K_ir_, and NaP channels in mediating the contrasting TBF-induced plasticity profiles in sub- vs. supra-threshold changes in DG granule cells.

### Activity-dependent intrinsic plasticity is mediated by calcium influx through synergistic interactions between *L*-type calcium channels and inositol trisphosphate receptors

**Figure 8:**
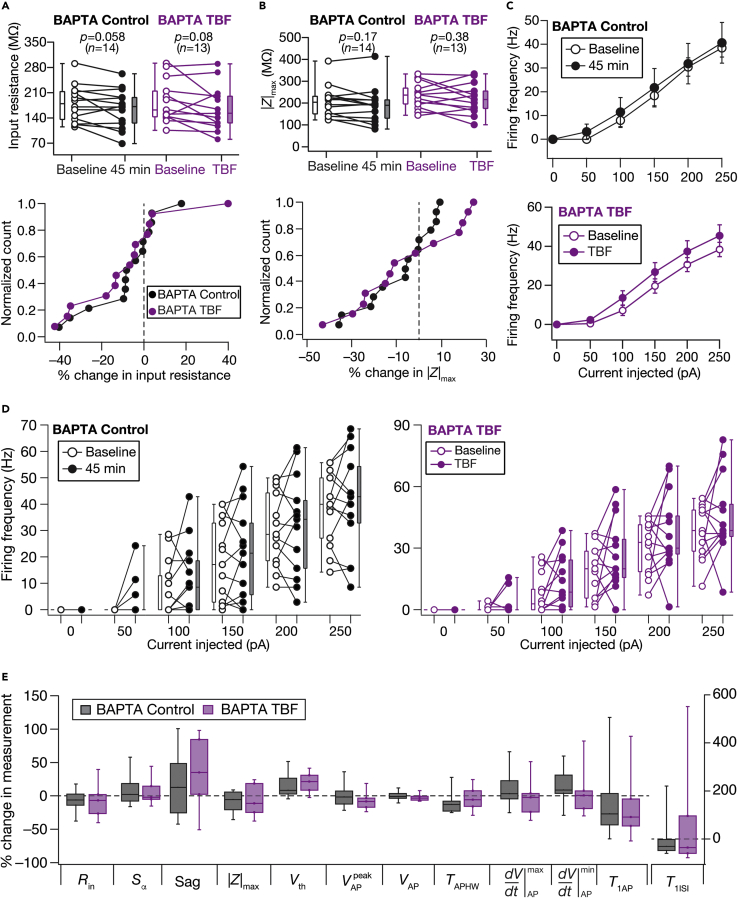
Activity-dependent intrinsic plasticity was dependent on calcium influx into the cytosol Control (black) and TBF (purple) group experiments were performed in the presence of 30-mM BAPTA in the pipette. The Control group corresponds to experiments where no protocol was applied through the 45-min period of the experiment, and the TBF group is for neurons subjected to TBF (A and B) Top: population data representing change in *R*_in_ (A) and |*Z*|_max_ (B) at the beginning (empty circles) and end (filled circles) of the experiment. Bottom: normalized count of neurons from the respective top panel plotted as functions of percentage change in *R*_in_ (A) and |*Z*|_max_ (B). Two-way mixed ANOVA, interaction p = 0.680 (*R*_in_) and 0.992 (|*Z*|_max_) (C) Summary statistics (mean ± SEM) of action potential firing frequency plotted as functions of injected current amplitude for both groups. ∗p <0.05; ∗∗p <0.005. Student’s *t* test (D) Population data representing changes in the action potential firing frequency at the beginning (empty circles) and end (filled circles) of the experiment, for six values of current injection, for the Control (left) and TBF (right) groups For each of the five (50, 100, 150, 200, and 250 pA) current injections, there was no significant interaction between time (0 vs. 45 min) and protocol (Control vs. TBF) factors when assessed with two-way mixed ANOVA. (E) Plots comparing percentage change in various measurements from their baseline-to-final values are provided for each measurement. The Wilcoxon signed rank test was used for *p*-value calculation in panels A and B, for comparing measurements from the same set of cells. The Wilcoxon rank-sum test was employed for p value calculation in panel E, to compare percentage changes in the Control vs. TBF group

As several forms of neuronal plasticity are dependent on cytosolic calcium influx, we performed a set of long-term Control and TBF experiments in the presence of 30-mM BAPTA, a fast calcium chelator, in the patch pipette. We observed no significant activity-dependent change in any of the sub- or supra-threshold measurements with either the control or the TBF experiments in the presence of BAPTA (Figure 8). We noticed that the overall firing rate of the cell was significantly high in the presence of BAPTA in the pipette in both control as well as TBF experiments (Figures 8C and 8D; *cf*. Figure 3), pointing to a critical role of calcium-activated potassium channels in suppressing the excitability of DG granule cells (Mateos-Aparicio et al., 2014).

**Figure 9:**
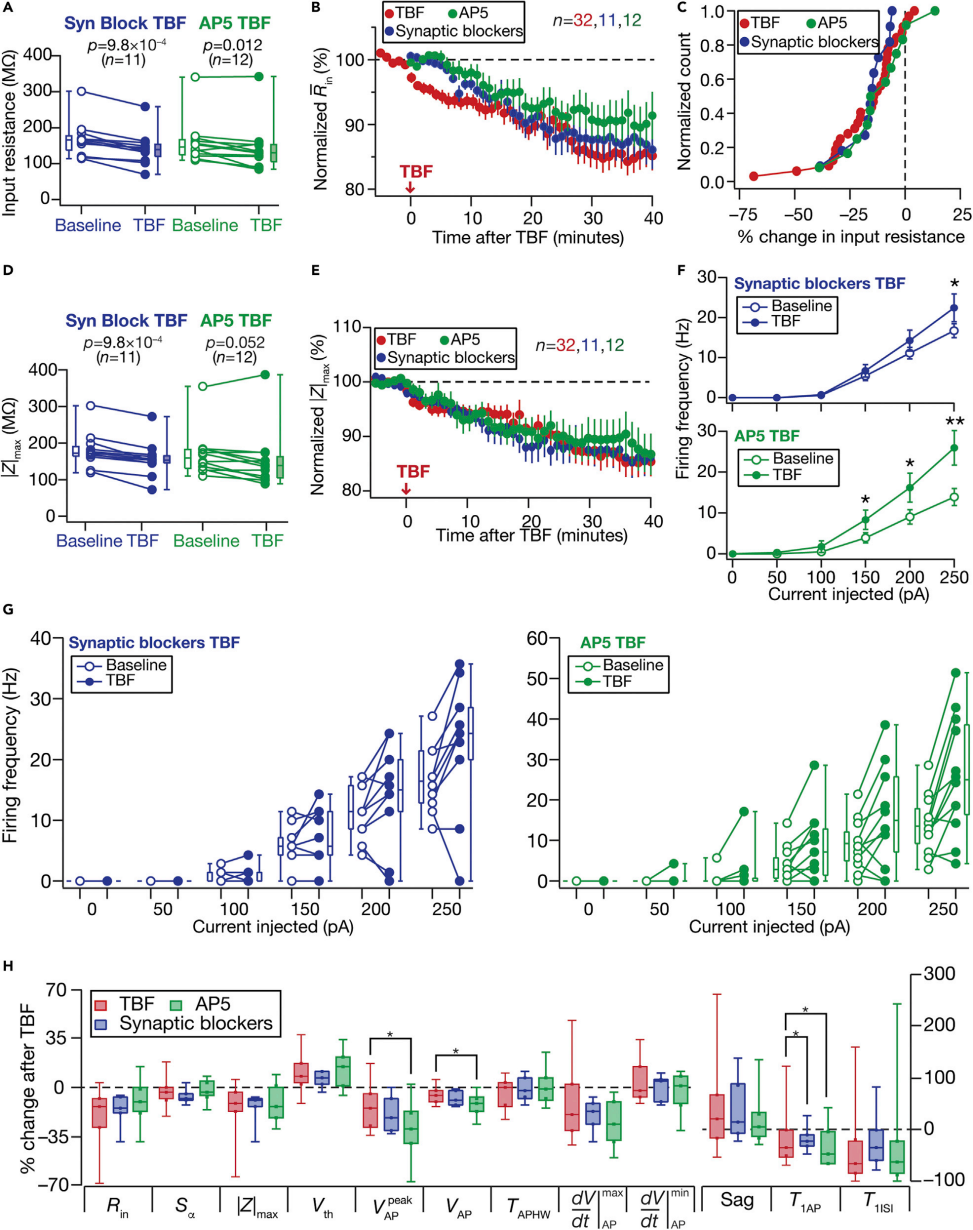
Activity-dependent intrinsic plasticity was independent of Glutamate and GABA receptors TBF experiments were performed in the presence of respective pharmacological agent(s) for the two groups: “Syn Block” (blue) represents blockers of AMPAR, GABA_A_R, and GABA_B_R; “AP5” (green) represents 50-μM AP5 in the bath (A) Population data representing change in *R*_in_ at the beginning (empty circles) and end (filled circles) of the experiment (B) Temporal evolution of percentage changes in ${\bar{R}}_{in}$ (mean ± SEM). Shown (red) for comparison is the time course for TBF experiments performed in normal ACSF from Figure 2B (C) Normalized count of neurons from panel A plotted as functions of percentage change in *R*_in_. Data from TBF experiments performed in normal ACSF from Figure 2C is shown for comparison (red) (D and E) Same as panels A–B, representing |*Z*|_max_ measurements. Shown in E (red) for comparison is the time course for TBF experiments performed in normal ACSF from Figure 2E (F) Summary statistics (mean ± SEM) of action potential firing frequency plotted as a function of injected current amplitude for both groups. ∗p <0.05; ∗∗p <0.005. Student’s *t* test (G) Population data representing changes in the action potential firing frequency at the beginning (empty circles) and end (filled circles) of the experiment, for six values of current injection, for the “Syn Block” (left) and the “AP5” (right) groups (H) Plots comparing percentage change in various measurements from their baseline-to-final values are provided for each measurement. Also shown (E) are percentage changes in respective measurements for TBF experiments performed in normal ACSF from Figures 2F and 3E. The Wilcoxon signed rank test was used for *p*-value calculation in panels A and D, for comparing measurements from the same set of cells. The Wilcoxon rank-sum test was employed for *p*-value calculation in panel H, to compare percentage changes in the Control vs. TBF group

**Figure 10:**
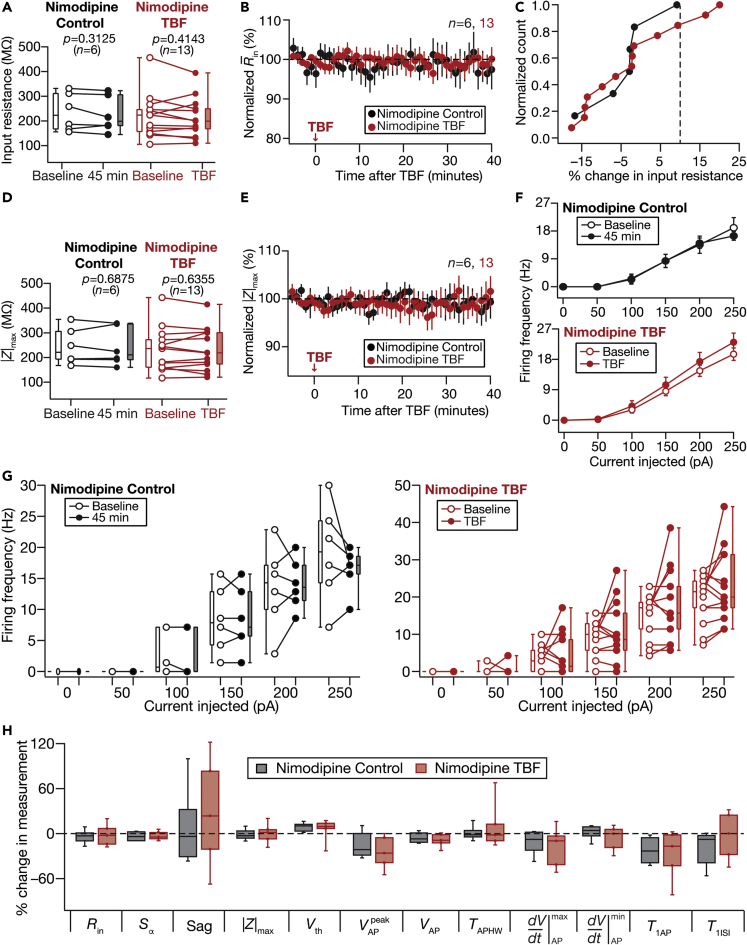
Activity-dependent intrinsic plasticity was dependent on calcium influx into the cytosol through *L*-type calcium channels Control (Black) and TBF (red) group experiments were performed in the presence of 10-μM Nimodipine in the bath. The Control groups (black) correspond to experiments where no protocol was applied through the 45-min period of the experiment, and TBF group (red) is for neurons subjected to TBF, with all experiments performed in the presence of Nimodipine. (A) Population data representing change in *R*_in_ at the beginning (empty circles) and end (filled circles) of the experiment. Two-way mixed ANOVA, interaction p = 0.897 (B) Temporal evolution of percentage changes in ${\bar{R}}_{\text{in}}$ (mean ± SEM) (C) Normalized count of neurons from panel A plotted as functions of percentage change in *R*_in_ (D and E) Same as panels A–B, representing |*Z*|_max_ measurements. Two-way mixed ANOVA, interaction p = 0.917 (F) Summary statistics (mean ± SEM) of action potential firing frequency plotted as a function of injected current amplitude for both groups. ∗p <0.05; ∗∗p <0.005. Student’s *t* test (G) Population data representing changes in the action potential firing frequency at the beginning (empty circles) and end (filled circles) of the experiment, for six values of current injection, for the Control (left) and TBF (right) groups. For each of the five (50, 100, 150, 200, and 250 pA) current injections, there was no significant interaction between time (0 vs. 45 min) and protocol (Control vs. TBF) factors when assessed with two-way mixed ANOVA (H) Plots comparing percentage change in various measurements from their baseline-to-final values are provided for each measurement. The Wilcoxon signed rank test was used for p value calculation in panels A and D, for comparing measurements from the same set of cells. The Wilcoxon rank-sum test was employed for p-value calculation in panel H, to compare percentage changes in the Control vs. TBF group

Although the TBF protocol does not involve stimulation of synaptic receptors, it is possible that different receptors driven by spontaneous activity could have interacted with intrinsic firing to play a role in plasticity induction (Fan et al., 2005). To test this, we performed TBF experiments in the presence of AMPAR, GABA_A_R, and GABA_B_R antagonists or NMDAR blockers, and found that the presence of these receptor blockers did not alter the expression of TBF-induced plasticity (Figure 9). With NMDARs ruled out as a calcium source, we hypothesized that repetitive firing triggered by the TBF could recruit voltage-gated calcium channels (VGCCs) as a source for calcium influx. To test this, we performed long-term Control and TBF experiments in the presence of nimodipine, a blocker of *L*-type VGCCs. We found that TBF-induced changes in both sub- and supra-measurements were abolished in the presence of nimodipine (Figure 10).

**Figure 11:**
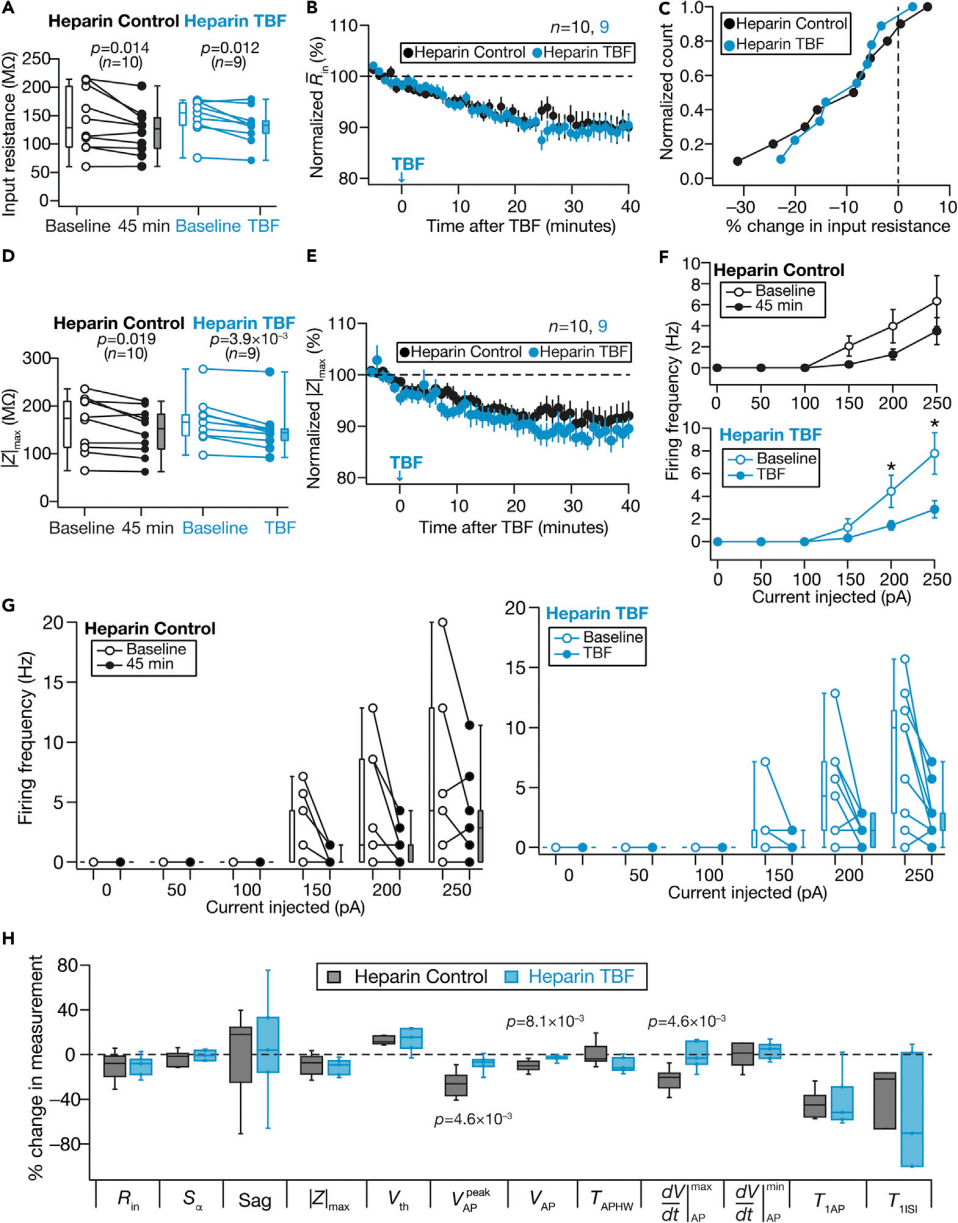
Activity-dependent intrinsic plasticity was dependent on calcium influx into the cytosol through inositol trisphosphate receptors Control (Black) and TBF (cyan) group experiments were performed in the presence of 1 mg/mL Heparin in the pipette. The Control groups (black) correspond to experiments where no protocol was applied through the 45-min period of the experiment, and TBF group (cyan) is for neurons subjected to TBF, with all experiments performed in the presence of Heparin (A) Population data representing change in *R*_in_ at the beginning (empty circles) and end (filled circles) of the experiment. Two-way mixed ANOVA, interaction *p* = 0.698 (B) Temporal evolution of percentage changes in ${\bar{R}}_{in}$ (mean ± SEM) (C) Normalized count of neurons from panel A plotted as functions of percentage change in *R*_in_ (D and E) Same as panels A–B, representing |*Z*|_max_ measurements. Two-way mixed ANOVA, interaction p = 0.701 (F) Summary statistics (mean ± SEM) of action potential firing frequency plotted as a function of injected current amplitude for both groups. ∗p <0.05; ∗∗p <0.005. Student’s *t* test (G) Population data representing changes in the action potential firing frequency at the beginning (empty circles) and end (filled circles) of the experiment, for six values of current injection, for the Control (left) and the TBF (right) groups. For each of the five (50, 100, 150, 200, and 250 pA) current injections, there was no significant interaction between time (0 vs. 45 min) and protocol (Control vs. TBF) factors when assessed with two-way mixed ANOVA (H) Plots comparing percentage change in various measurements from their baseline-to-final values are provided for each measurement. The Wilcoxon signed rank test was used for *p*-value calculation in panels A and D for comparing measurements from the same set of cells. The Wilcoxon rank-sum test was employed for *p*-value calculation in panel H, to compare percentage changes in the Control vs. TBF group

Finally, cytosolic calcium influx could also be through calcium channels on the ER membrane. Motivated by the expression of inositol trisphosphate (InsP_3_) receptors in DG granule cells (Fotuhi et al., 1993; Hertle and Yeckel, 2007; Nicolay et al., 2007; Sharp et al., 1993), we performed Control and TBF experiments in the presence of heparin, an InsP_3_-receptor blocker. We found that TBF-induced changes in various sub- and supra-threshold measurements were abolished in the presence of heparin, providing evidence for ER calcium stores as a potential source for plasticity induction (Figure 11).

Together, these experiments established that synergistic interactions between *L*-type VGCCs on the plasma membrane and InsP_3_ receptors on the ER membrane resulted in cytosolic calcium influx that mediated TBF-induced intrinsic plasticity. These experiments also suggest TBF-induced influx of calcium through *L*-type VGCCs as a source for initiating ER calcium release through InsP_3_ receptors (Ross, 2012).

## Discussion

We showed that the behaviorally relevant TBF protocol reliably induced long-term intrinsic plasticity in DG granule cells. We demonstrated that this intrinsic plasticity involved contrasting changes in sub- and supra-threshold excitability, inferred from a reduction in sub-threshold excitability that was not accompanied by a significant enhancement in action potential firing. Through a combination of experiments involving pharmacological agents and analyses of physiological changes induced by TBF, we provide strong lines of evidence for this form of plasticity to involve conjunctive changes in multiple ion channels. Specifically, we presented evidence that synergistic interactions between plasticity in HCN and K_ir_ channels mediated TBF-induced reduction in sub-threshold excitability whereas plasticity in NaP channels resulted in the accompanying increase in supra-threshold excitability. Finally, we showed that TBF-induced intrinsic plasticity was dependent on calcium influx through *L*-type VGCCs and InsP_3_ receptors.

### Activity-dependent intrinsic plasticity in DG granule cells and engram cell formation

Our study demonstrates that DG granule cells can express activity-dependent intrinsic plasticity *without* synaptic stimulation. The expression of cell-autonomous intrinsic plasticity expands the available repertoire of cellular substrates for information storage, from the perspective of what can change in response to activity patterns (Kim and Linden, 2007; Lopez-Rojas et al., 2016; Rathour and Narayanan, 2019; Titley et al., 2017; Mishra and Narayanan, 2021b; Zhang and Linden, 2003). Such neuron-specific enhancement of excitability constitutes the core of engram cell formation, whereby sparse neurons that were recruited during a specific behavioral context mediate encoding of that context through increased excitability (Gallistel, 2017; Pignatelli et al., 2019; Rao-Ruiz et al., 2019; Titley et al., 2017; Tonegawa et al., 2018).

**Figure 12:**
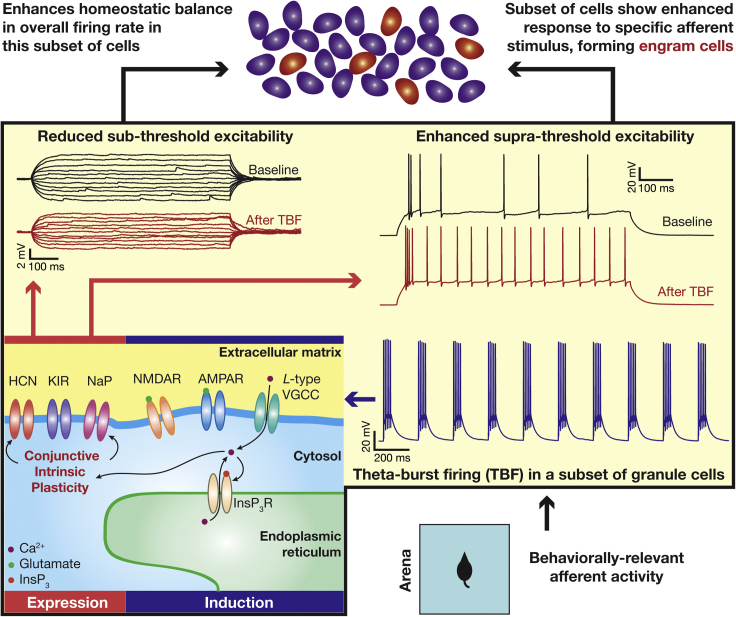
The expression of and implications for activity-dependent plasticity manifolds Behavioral context-dependent afferent inputs to the DG recruit a subset of granule cells, which elicit action potentials in theta-burst patterns. The findings from our study (within yellow box) show that TBF reduces sub-threshold excitability, but contrastingly increases supra-threshold excitability. Mechanistically, *induction* of this form of plasticity is dependent on cytosolic calcium influx through *L*-type VGCCs and InsP_3_Rs. The *expression* of TBF-induced contrasting plasticity in sub- and supra-threshold excitability is an outcome of conjunctive plasticity in multiple ion channels: HCN, K_ir_, and NaP. The enhanced supra-threshold excitability in the subset of contextually recruited cells (which manifested TBF) could result in the emergence of “engram cells” (highlighted in red in the top panel of granule cells), which *encode* for this specific behavioral context. The concomitant reduction in sub-threshold excitability, on the other hand, provides overall *homeostatic balance* in this subset of cells, to counteract the enhanced firing and enable *stable encoding* in this subset of granule cells.

It is well established that context-dependent afferent inputs to the DG activate a subset of granule cells, which elicit action potentials in theta-burst patterns (Diamantaki et al., 2016; Pernia-Andrade and Jonas, 2014; Zhang et al., 2020). Such sparse recruitment of neurons during specific behavioral contexts has been shown to rely on several factors, including targeted connectivity driven by adult neurogenesis, local inhibitory circuits, and heterogeneities in the excitability of granule cells (Aimone et al., 2014; Dieni et al., 2013; Josselyn and Frankland, 2018; Li et al., 2017; Lodge and Bischofberger, 2019; Mishra and Narayanan, 2020; Pignatelli et al., 2019; Rao-Ruiz et al., 2019; Yiu et al., 2014). Our study demonstrates that behaviorally observed theta-burst pattern of action potentials enhances the ability of neurons to fire more action potentials, specifically in those neurons that were recruited by afferent activity to elicit such patterns of activity. We postulate that the activity-dependent increase in supra-threshold excitability, along with other forms of plasticity, could provide a mechanistic basis for the emergence of engram cells that encode for a given context (Figure 12).

In this context, the manifestation of cell-to-cell variability (Figures 2, 3, and 4) in activity-dependent intrinsic plasticity could form a cellular substrate for why certain neurons (and not others) are primed to become engram cells (Josselyn and Frankland, 2018; Pignatelli et al., 2019; Rao-Ruiz et al., 2019; Yiu et al., 2014). Specifically, across measurements, whereas certain neurons showed tremendous changes in response to the same activity pattern, others did not (Figures 2, 3, and 4). In addition, expression of TBF-induced enhancement of supra-threshold excitability was reliant on a *competition* between two opposing changes (in sub- vs. supra-threshold excitability). We postulate that the sparse and orthogonal connectivity patterns in the DG, in conjunction with such competition among plasticity in different components, could play a critical role in regulating resource allocation for memory storage (Josselyn and Frankland, 2018; Li et al., 2017; Lodge and Bischofberger, 2019; Mishra and Narayanan, 2021b; Rao-Ruiz et al., 2019).

With specific reference to K_ir_ channel plasticity, (Pignatelli et al., 2019) investigated the dynamics of excitability of DG engram cells upon their reactivation by recall cues after contextual fear conditioning. Upon reactivation of DG engram cells, the authors found a transient reduction in K_ir_ currents associated with enhanced neural excitability in engram cells, immediately after recall. These experiments provide evidence for a role for K_ir_ channel plasticity in engram cells. However, there are differences in terms of the direction of changes, long-term vs. transient changes, and plasticity during encoding vs. recall with reference to our postulate here on the role of intrinsic plasticity in engram formation (Figure 12), thus precluding direct comparisons.

### Conjunctive plasticity in multiple ion channels points to plasticity manifolds: plasticity-stability balance

We demonstrated that the *same* plasticity protocol induces conjunctive plasticity in different channels. We argue that such conjunctive plasticity points to the presence of a tightly regulated *plasticity manifold*, whereby changes to different cellular components are not arbitrary but follow specific rules enforced by common or coupled molecular signaling cascades regulating such plasticity (Mishra and Narayanan, 2021b).

There are advantages of tightly regulated plasticity manifolds involving conjunctive plasticity in different structural components. For instance, our study presents a possible cell-autonomous route that a neuron could pursue to concomitantly achieve homeostasis while encoding or storage of information (Figure 12). Specifically, from the perspective of engram formation, we had earlier postulated that the activity-dependent increase in firing rate could assist the formation of engram cells that encode for a given context. Within such a postulate, the concomitant reduction in sub-threshold excitability would ensure that the response of the cell to other behavioral contexts is suppressed, apart from maintaining homeostatic balance (to compensate for the enhanced firing rate of the cell). Thus, the *contrasting* patterns of plasticity in sub- vs. supra-threshold excitability could be a cell-autonomous substrate to encode new information through enhanced excitability, accompanied by a mechanism to enhance specificity to individual contexts and to enable homeostatic balance of overall afferent drive to the neuron (Nelson and Turrigiano, 2008).

Our results are also reminiscent of plasticity manifolds in CA1 pyramidal neurons, whereby the *same* theta-burst pairing protocol induces putative *mnemonic* changes in synaptic strength (Magee and Johnston, 1997), in transient potassium channels (Frick et al., 2004; Losonczy et al., 2008), and in SK channels (Lin et al., 2008), apart from concomitantly inducing putative *homeostatic* changes in HCN channels (Fan et al., 2005; Honnuraiah and Narayanan, 2013; Narayanan and Johnston, 2007, 2010). In our study, we show that the *mnemonic* role could be played by plasticity in NaP channels and the *homeostatic* part could be played by changes in HCN and K_ir_ channels. We postulate that these conjunctive intrinsic plasticity mechanisms, along with synaptic plasticity, could also form ideal substrates for selective routing of spatial information flow (Zhang et al., 2020) and for maintaining the plasticity-stability balance in engram cells (Mishra and Narayanan, 2021b). Plasticity mechanisms presented here could subserve such roles by enhancing neuronal firing in specific contexts or at specific spatial locations while suppressing others. Thus, conjunctive plasticity involving multiple neural components forms an ideal substrate for effectively achieving the twin goal of encoding and homeostasis (Rathour and Narayanan, 2019) in a cell-type dependent manner. The strong rules governing *concomitant plasticity in multiple components* ensure that the encoding and homeostasis processes don’t interfere with each other, thereby avoiding catastrophic forgetting or unstable learning and enabling continual stable learning, also providing a mechanistic basis for population activity to remain within a neuronal activity manifold (Mishra and Narayanan, 2021b).

### Activity-dependent intrinsic plasticity, metaplasticity, and channelopathies

It is now well established that plasticity in intrinsic properties could result in metaplasticity through changes in excitability and in temporal summation (Anirudhan and Narayanan, 2015; Hulme et al., 2013; Narayanan and Johnston, 2010; Sehgal et al., 2013). Our results, demonstrating changes to temporal summation and sub- and supra-threshold excitability would therefore result in metaplasticity in plasticity profiles of the DG. Such metaplasticity introduced by intrinsic plasticity could form a putative substrate for changes in threshold for synaptic plasticity by *prior* theta-frequency synaptic activity (Abraham, 2008; Christie et al., 1995). From a pathophysiological perspective, altered activity patterns have been associated with intrinsic plasticity in the DG, including in channels explored in this study (Beck and Yaari, 2008; Bender et al., 2003; Stegen et al., 2009, 2012; Surges et al., 2012; Yim et al., 2015; Young et al., 2009). Specifically, conjunctive changes in HCN and K_ir_ channels reported in this study are reminiscent of changes in these two channels observed in human DG granule cells with temporal-lobe epilepsy (Stegen et al., 2012). Therefore, the cellular and ion channel mechanisms associated with intrinsic plasticity explored in this study could play a role in the presence of pathological activity patterns as well.

### Limitations of the study

Although we do not know the specific reasons for a time-dependent reduction in *R*_in_ in the presence of riluzole (Figure 7), it could be a consequence of the action of riluzole on one of its several known targets (Bryson et al., 1996; Dimitriadi et al., 2013; Doble, 1996; Duprat et al., 2000; Frizzo et al., 2004; Fumagalli et al., 2008). Among these is the ability of riluzole to activate two-pore domain potassium channels, targeting specific subunits (Duprat et al., 2000) that are known to express and alter excitability of DG granule cells (Reyes et al., 2000; Yarishkin et al., 2014). Such activation of two-pore domain potassium channels could potentially explain the time dependent reduction in *R*_in_ we observed. In addition, there are known non-specificities of ZD7288 (Chen, 2004; Chevaleyre and Castillo, 2002; Sanchez-Alonso et al., 2008), Ba^2+^ (Zhou et al., 2012), which constitute well-established limitations of the use of pharmacological agents (discussed in the context of DG granule cells in (Mishra and Narayanan, 2021a)). However, in our study, we have also employed a constellation of physiological measurements showing signature physiological changes (Figures 1, 2, 3, and 4) to provide lines of evidence for roles of the different ion channels in the reported form of plasticity. Future experiments need to couple cell-attached recordings targeting specific ion-channel currents along with theta-burst firing to probe the role of individual ionic currents and ion-channel gating properties in mediating TBF-induced plasticity (*e*.*g*. (Frick et al., 2004)).

Although our focus in this study was limited to the somata of DG granule cells and on three specific ion channels, it is possible that other channels might change in response to TBF and the manifestation of plasticity might be distinct at different somato-dendritic locations. Therefore, future studies should assess plasticity in other channels employing recordings spanning the somato-dendritic axis of DG granule cells. Such analyses would provide the complete span of the plasticity manifold with reference to this form of intrinsic plasticity. Future studies should also explore the roles of metabotropic receptors, ER components, and signaling cascades downstream of calcium elevation (including the role of different kinases and phosphatases, using specific enzyme inhibitors) that mediate conjunctive plasticity in these ion channels.

Our recordings for this study were limited to the crest region of the dentate gyrus from the middle hippocampi (in the dorsoventral axis). However, there could be differences in TBF-induced plasticity expression in other subregions of the DG. Future studies should therefore explore TBF-induced intrinsic plasticity across the dorsoventral, superficial-deep and infrapyramidal-supra-pyramidal axes of the DG (Amaral et al., 2007) to assess potential differences in plasticity expression across different subregions of the DG. Furthermore, although there are several studies exploring differences in synaptic plasticity profiles in mature vs. immature neurons of the DG (Aimone et al., 2011, 2014; Dieni et al., 2013; Kropff et al., 2015; Li et al., 2017; Schmidt-Hieber et al., 2004), differences in intrinsic plasticity profiles in mature vs. immature neurons has not been explored. These differences might play a role in how immature neurons integrate into a functional network, and how they participate in engram formation with reference to a specific context. Additionally, as the impact of neuromodulatory inputs on intrinsic plasticity profiles has not been explored, future studies should explore the role of adult neurogenesis and neuromodulators in induction and expression of intrinsic plasticity.

## STAR★Methods

### Key resources table

**Table:** 

REAGENT or RESOURCE	SOURCE	IDENTIFIER
**Chemicals, Peptides, and Recombinant Proteins**		

Sodium Chloride	Sigma Aldrich (Merck)	Cat#: S5886 CAS: 7647-14-5
Potassium Chloride	Sigma Aldrich (Merck)	Cat#: P5405 CAS: 7447-40-7
Potassium Gluconate	Sigma Aldrich (Merck)	Cat#: G4500 CAS: 299-27-4
Sodium Bicarbonate	Sigma Aldrich (Merck)	Cat#: S5761 CAS: 144-55-8
HEPES	Sigma Aldrich (Merck)	Cat#: H3375 CAS: 7365-45-9
Dextrose	Sigma Aldrich (Merck)	Cat#: D9434 CAS: 50-99-7
Sucrose	Sigma Aldrich (Merck)	Cat#: S9378 CAS: 57-50-1
Barium Chloride	Sigma Aldrich (Merck)	Cat#: 342920 CAS: 10361-37-2
Magnesium Chloride Hexahydrate	Sigma Aldrich (Merck)	Cat#: M2393 CAS: 7791-18-6
Calcium Chloride Dihydrate	Sigma Aldrich (Merck)	Cat#: C5080 CAS: 10035-04-8
Magnesium ATP	Sigma Aldrich (Merck)	Cat#: A9187 CAS: 74804-12-9
Sodium GTP	Sigma Aldrich (Merck)	Cat#: G8877 CAS: 36051-31-7
Sodium Pyruvate	Sigma Aldrich (Merck)	Cat#: P5280 CAS: 113-24-6
Potassium Hydroxide	Sigma Aldrich (Merck)	Cat#: 306568 CAS: 1310-58-3
Sodium phosphate monobasic monohydrate	Sigma Aldrich (Merck)	Cat#: S3522 CAS: 10049-21-5
K2 Phosphocreatine	Sigma Aldrich (Merck)	Cat#: 237911 CAS: 18838-38-5
Picrotoxin	Abcam	Cat#: ab120315 CAS: 124-87-8
6-cyano- 7-nitroquinoxaline-2,3-dione (CNQX)	Abcam	Cat#: ab120044 CAS: 479347-85-8
d,l-2-amino-5-phosphonovaleric acid (d,l-APV)	Abcam	Cat#: ab120271 CAS: 1303993-72-7
Bicuculine	Abcam	Cat#: ab120107 CAS: 485-49-4
ZD7288	Abcam	Cat#: ab120102 CAS: 133059-99-1
Riluzole	Abcam	Cat#: ab120272 CAS: 850608-87-6
Tetrapotassium BAPTA	Thermofisher	Cat#: B1204 CAS: 73630-08-7
Nimodipine	Tocris	Cat#: 0600 CAS: 66085-59-4
Heparin	Calbiochem (Merck)	Cat#: 375095 CAS: 9041-08-1
Tertiapin-Q	Tocris	Cat#: 1316 CAS: 910044-56-3
CGP 55845 hydrochloride	Abcam	Cat#: ab120337 CAS: 149184-22-5

**Experimental Models: Organisms/Strains**		

Sprague Dawley rat	Central Animal Facility, Indian Institute of Science, Bangalore	RRID: MGI:5651135

**Software and Algorithms**		

Igor Pro	Proprietary software	http://wavemetrics.com
Electrophysiology acquisition & Analysis software	(Narayanan and Johnston, *Neuron*, 2007; Narayanan and Johnston, *J Neuroscience, 2008*; Mishra and Narayanan, *J Neurophysiology, 2020*)	N/A

### Resource availability

#### Lead contact

Further information and requests for resources and reagents should be directed to and will be fulfilled by the Lead Contact, Rishikesh Narayanan (rishi@iisc.ac.in).

#### Materials availability

This study did not generate new unique reagents.

### Experimental model and subject details

This *in vitro* electrophysiological study employed male Sprague-Dawley rats of 6- to 8-weeks age. All experiments reported in this study were performed in strict adherence to the protocols approved by the Institute Animal Ethics Committee (IAEC) of the Indian Institute of Science, Bangalore. Animals were provided *ad libitum* food and water and were housed with an automated 12 h light–12 h dark cycle. All animals were obtained from the in-house breeding setup at the central animal facility of the Indian Institute of Science.

### Method details

Surgical and electrophysiological procedures were similar to previously established protocols (Ashhad and Narayanan, 2016; Mishra and Narayanan, 2020; Narayanan and Johnston, 2007, 2008) and are detailed below.

#### Slice preparation for *in-vitro* patch clamp recording

Rats were anesthetized by intraperitoneal injection of a ketamine-xylazine mixture. After onset of deep anesthesia, assessed by cessation of toe-pinch reflex, transcardial perfusion of ice-cold cutting solution was performed. The cutting solution contained 2.5 mM KCl, 1.25 mM NaH_2_PO_4_, 25 mM NaHCO_3_, 0.5 mM CaCl_2_, 7 mM MgCl_2_, 7 mM dextrose, 3 mM sodium pyruvate, and 200 mM sucrose (pH 7.3, ∼300 mOsm) saturated with 95% O_2_ and 5% CO_2_. Thereafter, the brain was removed quickly and 350-μm thick near-horizontal slices were prepared from middle hippocampi (Bregma, −6.5 mm to −5.1 mm), using a vibrating blade microtome (Leica Vibratome), while submerged in ice-cold cutting solution saturated with 95% O_2_ and 5% CO_2_. The slices were then incubated for 10–15 min at 34°C in a chamber containing the holding solution (pH 7.3, ∼300 mOsm) with the composition of: 125 mM NaCl, 2.5 mM KCl, 1.25 mM NaH_2_PO_4_, 25 mM NaHCO_3_, 2 mM CaCl_2_, 2 mM MgCl_2_, 10 mM dextrose, 3 mM sodium pyruvate saturated with 95% O_2_ and 5% CO_2_. The slices were kept in a holding chamber at room temperature for at least 45 min before the start of recordings.

#### Electrophysiology: whole-cell current-clamp recording

For electrophysiological recordings, slices were transferred to the recording chamber and continuously perfused with carbogenated artificial cerebrospinal fluid (ACSF/extracellular recording solution) at a flow rate of 2–3 mL/min. All neuronal recordings were performed under current-clamp configuration at physiological temperatures (32–35°C), achieved through an inline heater that was part of a closed-loop temperature control system (Harvard Apparatus). The carbogenated ACSF contained 125 mM NaCl, 3 mM KCl, 1.25 mM NaH_2_PO_4_, 25 mM NaHCO_3_, 2 mM CaCl_2_, 1 mM MgCl_2_, 10 mM dextrose (pH 7.3; ∼300 mOsm). Slices were first visualized under a 10× objective lens to locate the granule cell layer in the crest sector of the dentate gyrus (Amaral et al., 2007; Mishra and Narayanan, 2020). Then, a 63× water immersion objective lens was employed to perform patch-clamp recordings from DG granule cells in the crest sector, through a Dodt contrast microscope (Carl Zeiss Axioexaminer). Whole-cell current-clamp recordings were performed from visually identified dentate gyrus granule cell somata, using Dagan BVC-700A amplifiers. Electrophysiological signals were low-pass filtered at 5 kHz and sampled at 10–40 kHz. All data acquisition and analyses were performed using custom-written software in Igor Pro (Wavemetrics).

Images of cell location were captured for record and for post-facto confirmation of the region where the cell belonged. Borosilicate glass electrodes with resistance between 2 and 6 MΩ (more often electrodes with ∼4 MΩ electrode resistance were used) were pulled (P-97 Flaming/Brown micropipette puller; Sutter) from thick glass capillaries (1.5 mm outer diameter and 0.86 mm inner diameter; Sutter) and used for patch-clamp recordings. The pipette solution contained 120 mM K-gluconate, 20 mM KCl, 10 mM HEPES, 4 mM NaCl, 4 mM Mg-ATP, 0.3 mM Na-GTP, and 7 mM K2-phosphocreatine (pH 7.3 adjusted with KOH, osmolarity ∼300 mOsm).

Series resistance was monitored (once every 30 s) and compensated online using the bridge-balance circuit of the amplifier. Experiments were discarded only if the initial resting membrane potential was more depolarized than −60 mV or if series resistance rose above 30 MΩ, or if there were fluctuations in temperature and ACSF flow rate during the course of the experiment. Unless otherwise stated, experiments were performed at the initial resting membrane potential (reported here as *V*_RMP_) of the cell. Voltages have not been corrected for the liquid junction potential, which was experimentally measured to be ∼8 mV.

#### Sub-threshold measurements

We characterized plasticity in intrinsic properties of DG granule neurons using several electrophysiological measurements obtained through several pulse-current and frequency-dependent current injections (Ashhad and Narayanan, 2016; Mishra and Narayanan, 2019; Narayanan and Johnston, 2007, 2008). Input resistance (*R*_in_) was measured as the slope of a linear fit to the steady-state *V-I* plot obtained by injecting subthreshold current pulses of amplitudes spanning −25 to +25 pA, in steps of 5 pA (Figure 1D). To assess temporal summation, five α-excitatory postsynaptic potentials (α-EPSPs) with 50 ms interval were evoked by current injections of the form *I*_α_ = *I*_max_ *t* exp (–α*t*), with α = 0.1 ms^−1^ (Figure 1G). Temporal summation ratio (*S*_α_) in this train of five EPSPs was computed as *E*_last_/*E*_first_, where *E*_last_ and *E*_first_ were the amplitudes of last and first EPSPs in the train, respectively. Percentage sag was measured from the voltage response of the cell to a hyperpolarizing current pulse of 100 pA (Figures 1B, top) and was defined as 100 (1–*V*_ss_/*V*_peak_), where *V*_ss_ and *V*_peak_ depicted the steady-state and peak voltage deflection from *V*_RMP_, respectively.

The chirp stimulus (Figure 1B) used for characterizing the impedance amplitude (ZAP) profiles was a sinusoidal current of constant amplitude below firing threshold, with its frequency linearly spanning 0–15 Hz in 15 s (*Chirp15*). The magnitude of the ratio of the Fourier transform of the voltage response (Figure 1E) to the Fourier transform of the *Chirp15* stimulus formed the ZAP (Figure 1F):

|Z(f)|=(Re(Z(f)))2+(Im(Z(f)))2

where Re(*Z*(*f*)) and Im(*Z*(*f*)) refer to the real and imaginary parts of the impedance *Z* as a function of frequency *f*. The maximum value of impedance across all frequencies was measured as the maximum impedance amplitude (|*Z*|_max_; Figure 1F). The frequency at which the impedance amplitude reached its maximum was the resonance frequency (*f*_R_). Resonance strength (*Q*) was measured as the ratio of the maximum impedance amplitude to the impedance amplitude at 0.5 Hz (Narayanan and Johnston, 2007). As neurons seldom receive steady-state inputs under ethological conditions, impedance constitutes a better measure of excitability than measures based on pulse-current injections (Narayanan and Johnston, 2008).

#### Supra-threshold measurements

To understand the possible cellular mechanisms underlying the expression of activity-dependent intrinsic plasticity, we further analyzed the various features of action potentials. First, AP firing frequency was computed by extrapolating the number of spikes obtained during a 700 ms current injection to 1 s. Current amplitude of these pulse-current injections was varied from 0 pA to 250 pA in steps of 50 pA, to construct the firing frequency vs. injected current (*f–I*) plot (Figures 1H and 1I). Various AP related measurements (Mishra and Narayanan, 2019, 2020, 2021a) were derived from the voltage response of the cell to a 250 pA pulse-current injection. AP amplitude (*V*_AP_) was computed as the difference between the peak voltage of the spike $\left(V{\;}_{AP}^{\text{peak}}\right)$ and *V*_RMP_. The temporal distance between the timing of the first spike and the time of current injection was defined as latency to first spike (*T*_1AP_). The duration between the first and the second spikes was defined as the first inter-spike interval (*T*_1ISI_). AP half-width (*T*_APHW_) was the temporal width measured at the half-maximal points of the AP peak with reference to *V*_RMP_. The maximum ${\frac{\mathrm{d}V}{\mathrm{d}t}|}_{AP}^{\text{max}},\text{and}\;\text{minimum }{\frac{\mathrm{d}V}{\mathrm{d}t}|}_{AP}^{\text{min}}$ values of the AP temporal derivative were calculated from the temporal derivative of the AP trace. The voltage in the AP trace corresponding to the time point at which the d*V*/d*t* crossed 20 V/s defined AP threshold. All supra-threshold measurements were obtained through current injections into the cell resting at *V*_RMP_.

#### TBF protocol

Experimental procedures for inducing theta-burst firing (TBF) were similar to previously established protocols (Fan et al., 2005; Narayanan and Johnston, 2007). To induce TBF, we employed 3 trains of 10 theta-modulated bursts each, separated by 10 s. Each train (Figure 1C) was made of a burst of 5 APs with an intra-burst frequency of 100 Hz (10 ms ISI), and the inter-burst frequency set at 5 Hz (200 ms). Each AP within the burst was initiated by injecting a large current (2 nA) of small duration (2 ms) into the neuron. The experimental protocol (Figures 1B and 1C) for TBF involved initial baseline measurements of the *V-I* curve, *f-I* curve and *S*_α_, followed by establishment of a 5 min stable baseline while monitoring resting membrane potential and *R*_in_. Monitoring of these measurements were from cell responses to the *Chirp15* stimulus (Figure 1B), measured twice every minute, during this initial 5 min period and for 40 min after TBF, followed by a final measurement of the *V-I* curve, *f-I* curve and *S*_α_. A large 100-pA hyperpolarizing current pulse was provided before the chirp current to compute an input resistance estimate $\left({\bar{R}}_{in}\right)$ and to observe and correct series resistance changes through the course of the experiment. We recorded from one selected cell in any given slice, as the induction protocol could have had unknown effects on neighboring cells through synaptic transmission or other signaling cascades. To assess pairwise relationships across 13 different sub- and supra-threshold measurements, we analyzed the scatter plot matrices of post-TBF changes in these measurements. We computed Pearson’s correlation coefficients for each of these pair-wise scatter plots and analyzed the distribution of correlation coefficients (Figure 4A).

The impact of slow ion channels, such as HCN channels, on neuronal excitability for relatively high-frequency inputs is low, as the slow activation and deactivation profiles of these channels are inconsistent with such fast inputs. Therefore, changes (*e.g.,* plasticity, blockade) in such slow channels would have relatively larger impact on impedance amplitude (|*Z*(*f*)|) at lower frequencies. This characteristic has been employed (Narayanan and Johnston, 2008) as an effective mechanism to distinguish between changes in leak conductance (which would change excitability equally at all frequencies) or membrane capacitance (which would alter excitability preferentially at higher frequencies) or HCN channels (which would alter excitability preferentially at lower frequencies). To assess the impact of experimental protocols on slow ion channels, we computed changes in |*Z*| as a function of frequency (Figure 4E) and compared the area under the curve (AUC) of the |Z| vs. *f* plot in three frequency bands (0–5 Hz, 5–10 Hz an 10–15 Hz) across different experimental protocols (Figure 4F).

#### Pharmacological blockers

##### Synaptic receptor blockers

Drugs and their concentrations used in the experiments were 10 μM 6-cyano- 7-nitroquinoxaline-2,3-dione (CNQX), an AMPA receptor blocker; 10 μM (+) bicuculline and 10 μM picrotoxin, both GABA_A_ receptor blockers; 50 μM d,l-2-amino-5-phosphonovaleric acid (d,l-APV), NMDA receptor and 2 μM CGP55845, GABA_B_ blocker (all synaptic blockers from Abcam) in the bath solution. To block *Inositol trisphosphate (InsP*_*3*_*) receptors*, 1 mg/mL heparin (20,000–25,000 molecular weight; Calbiochem) was included in the recording pipette.

##### Voltage-gated ion channel blockers

20 μM ZD7288 (Abcam) was employed to block HCN channels. 50 μM BaCl_2_ (Sigma Aldrich) was used to block inward-rectifier potassium channels. 20 μM Riluzole (Abcam) and 10 μM Nimodipine (Tocris Biosciences) were used to block persistent sodium and *L*-type calcium channels, respectively. For experiments with ZD7288, cells were patched with pipette solution containing 20 μM ZD7288 along with adding it to bath solution (Ashhad and Narayanan, 2016). For long-term control and TBF experiments in the presence of pharmacological agents, slices were pretreated with the respective pharmacological agent for at least 15 min before the start of recordings.

##### Fast calcium chelator

30 mM (1,2-bis(o-aminophenoxy)ethane-N,N,N′,N'-tetraacetic acid), BAPTA (Thermo Fisher) was incorporated into the pipette solution. The constituents of the pipette solution were (in mM): 30 mM K_4_-BAPTA, 20 mM KCl, 10 mM HEPES, 4 mM NaCl, 4 mM Mg-ATP, 0.3 mM Na-GTP, and 7 mM K_2_-phosphocreatine (pH 7.3 adjusted with KOH, osmolarity ∼300 mOsm adjusted with sucrose).

### Quantification and statistical analysis

All statistical analyses were performed using the R computing package (http://www.r-project.org/). In order to avoid false interpretations and to emphasize the heterogeneities, the entire range of measurements are reported in figures rather than providing only the summary statistics (Rathour and Narayanan, 2019). Necessary care was taken and appropriate controls were performed for each of the drugs used to account for any time-dependent changes initiated by just the presence of the drug in the bath or in the pipette solution. For all cases, we performed long-term control experiments (no protocol) and TBF experiments in the presence of same quantity of drugs, and report the entire span of measurements corresponding to the outcomes for both sets of experiments. Statistical comparison was performed with their respective long-term controls using two-way mixed ANOVA with interaction between the “within” (measurements at 0 min vs. 45 min) and “between” (Control vs. TBF groups) cell factors. We took advantage of our experimental design that involved respective controls for all pharmacological agents, and employed mixed two-way ANOVA to assess all our results. Specifically, for each physiological measurement (input resistance, firing rate, impedance amplitude, etc.), we have two groups of cells (“Control” group, which did not undergo any protocol, and “TBF” group that underwent theta-burst firing protocol). For each of these two groups of cells, we have measurements that are compared at two time points (At the beginning of the experiment and the end of the experiment at 45 min). We now employed the protocol applied (Control vs. TBF) as the “between cells” factor and the time period as the “within cell” factor to perform a two-way mixed ANOVA on each measurement for each set of experiments performed. We report the interaction p value for the measurements in Tables S1 and S2 and/or in the respective legends. Across figures, the statistics employed for data presentation was consistent with the statistical test used to compare two populations of data. Specifically, when data is reported as mean ± SEM, parametric tests (paired or unpaired Student’s *t* test) were employed, and when data is reported as median (along with the entire distribution of the data or the quartiles), we employed non-parametric tests (Wilcoxon ranked sum or signed rank tests). Results of statistical tests, with exact p values and the name of the statistical test employed, are provided in the figure panels or in the respective figure legends (also see Tables S1 and S2).

## Data Availability

• The published article includes all datasets generated or analyzed during this study. All data reported in this paper will be shared by the lead contact upon request. • This paper did not report original code. • Any additional information required to reanalyze the data reported in this paper is available from the lead contact upon request.

## Supplemental information

Document S1. Figures S1, S2, Tables S1 and S2

Data S1. Data presented in each of Figures 2–11 is provided in a supplemental excel sheet, related to Figures 2–11 Different sheets in the Excel file contain data for each of these 10 figures
