# Supplementary material for: Conjunctive changes in multiple ion channels mediate activity-dependent intrinsic plasticity in hippocampal granule cells
Source: iScience. 2022 Feb 14;25(3):103922. doi: 10.1016/j.isci.2022.103922 (PMC8894279; doi:10.1016/j.isci.2022.103922)
Supplement: Document S1. Figures S1, S2, Tables S1 and S2 [file mmc1.pdf]

iScience, Volume *25*

Supplemental information

Conjunctive changes in multiple ion channels mediate activity-dependent intrinsic plasticity in hippocampal granule cells

Poonam Mishra and Rishikesh Narayanan

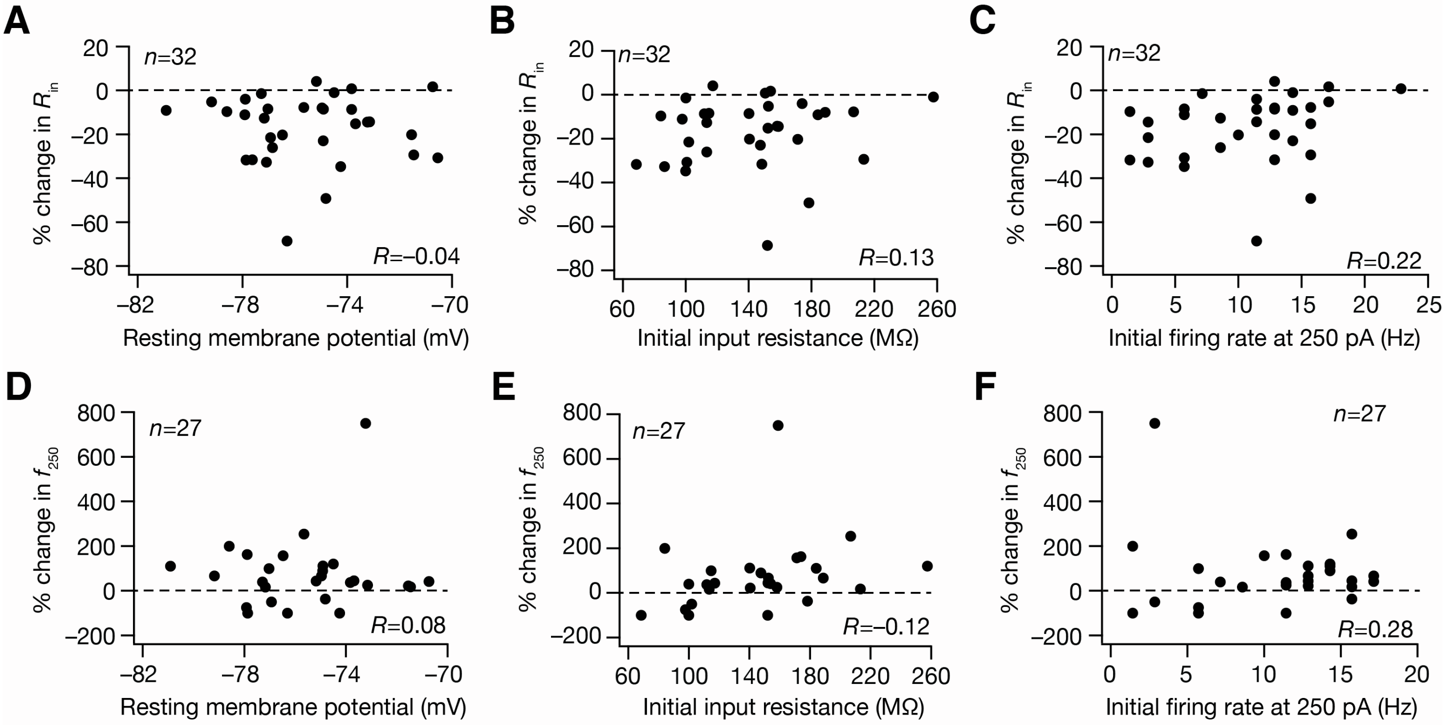

Supplemental Figure S1. Related to Figure 4

Weak correlations between heterogeneities in TBF-induced plasticity and heterogeneities in intrinsic properties of DG granule cells. ***A–C***, Population data representing TBF-induced change in input resistance plotted against resting membrane potential of the cells (***A***); input resistance of the cells (***B***); and firing rate of the cells in response to 250 pA current injection (***C***). ***D–F***, Same as (***A–C***), but with TBF-induced change in firing rate at 250 pA on the ordinate. Pearson correlation coefficient (*R*) is provided for each panel. The data for plasticity in sub- and supra-threshold measurements is from the TBF group of Fig. 2.

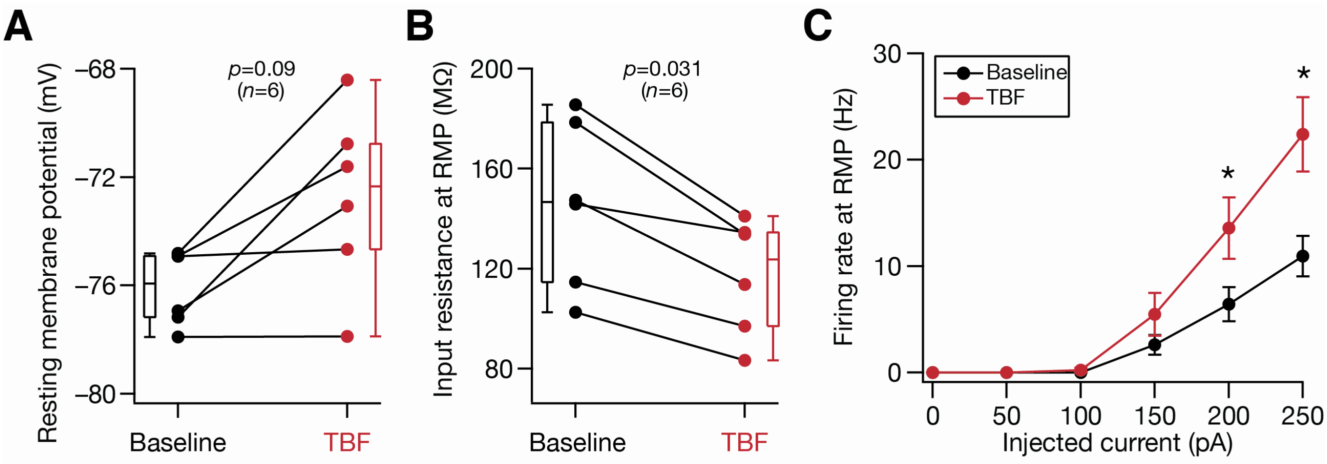

Supplemental Figure S2. Related to Figure 4

Contrasting plasticity in sub- *vs.* supra-threshold excitability was observed when measurements were recorded at respective resting membrane potentials. (A) Population data of resting membrane potential from all DG granule cells recorded before (black) and 40 mins after (red) TBF protocol, showing persistent changes in RMP after TBF. (B–C) Population data of measurements from all DG granule cells recorded before (black) and 40 mins after (red) TBF protocol. Measurements were performed at the respective resting membrane potentials (shown in panel A for each cell), with zero current injection. Input resistance obtained from the *V–I* plot reduced after TBF (B), whereas firing rate increased (C). The Wilcoxon signed rank test was employed for *p* value calculation in panels A–B, and Student's *t* test was employed for *p* value calculation in panel C. *: $p<0.05$.

Supplemental Table S1. Related to Figure 2

Sub-threshold physiological properties measured before and 40 minutes after TBF. These statistics correspond to the data plotted in Fig. 2. *p* values correspond to paired Student's *t* test, and data are represented as mean ± SEM.

	Measurement (units)	Control Group			TBF Group		
		**0 min**	**45 min**	***p* value (*t* test)**	**0 min**	**45 min**	***p* value (*t* test)**
1	Resting membrane potential, V_{RMP} (mV)	–75.6 ± 3.1	–75.6 ± 3.3	0.95	–75.9 ± 0.6	–72.3 ± 1.2	6.3×10^{-4}
2	Input resistance, R_{in} (MΩ)	152.8 ± 15.9	150.6 ± 14.7	0.56	141.8 ± 7.5	118.2 ± 8.0	4.4×10^{-6}
3	Maximal impedance amplitude, $\|Z\|_{max}$ (MΩ)	182.5 ± 28.9	186.3 ± 32.9	0.49	155.9 ± 8.2	134.2 ± 8.8	1.4×10^{-5}
4	Resonance frequency, f_R (Hz)	0.77 ± 0.05	0.71 ± 0.07	0.45	0.77 ± 0.01	0.99 ± 0.20	0.27
5	Resonance strength, Q	1.04 ± 0.01	1.03 ± 0.015	0.66	1.03 ± 0.03	1.03 ± 0.003	0.86
6	Total inductive phase, Φ_L (rad.Hz)	0.014 ± 0.009	0.02 ± 0.008	0.56	0.01 ± 0.002	0.01 ± 0.001	0.33
7	Sag (%)	3.56 ± 0.69	2.90 ± 0.49	0.40	2.6 ± 0.14	3.3 ± 0.31	0.037
8	Temporal summation ratio, S_α	1.37 ± 0.03	1.33 ± 0.04	0.38	1.3 ± 0.03	1.23 ± 0.02	3.5×10^{-3}

	Measurement (units)	*p* values from two-way mixed mode ANOVA		
		**Within cell factor (0 min *vs.* 45 min)**	**Between cells factor (Control *vs.* TBF)**	**Interaction**
1	Resting membrane potential, V_{RMP} (mV)	0.0022	0.606	0.0023
2	Input resistance, R_{in} (MΩ)	0.0023	0.176	0.0097
3	Maximal impedance amplitude, $\|Z\|_{max}$ (MΩ)	0.034	0.087	0.0032
4	Resonance frequency, f_R (Hz)	0.608	0.436	0.470
5	Resonance strength, Q	0.917	0.945	0.783
6	Total inductive phase, Φ_L (rad.Hz)	0.796	0.952	0.409
7	Sag (%)	0.956	0.503	0.062
8	Temporal summation ratio, S_α	0.0069	0.071	0.611

Supplemental Table S2. Related to Figure 3

Supra-threshold physiological properties measured before and 40 minutes after TBF. These statistics correspond to the data plotted in Fig. 3. *p* values correspond to paired Student's *t* test, and data are represented as mean ± SEM.

	**Measurement (units)**	**Control Group**			**TBF Group**		
		**0 min**	**45 min**	***p* value (*t* test)**	**0 min**	**45 min**	***p* value (*t* test)**
1	Firing frequency at 50 pA, f_{50} (Hz)	0 ± 0	0 ± 0	—	0 ± 0	0 ± 0	—
2	Firing frequency at 100 pA, f_{100} (Hz)	0 ± 0	0 ± 0	—	0.1 ± 0.07	0.95 ± 0.44	0.043
3	Firing frequency at 150 pA, f_{150} (Hz)	1.07 ± 0.59	1.42 ± 0.71	0.35	2.54 ± 0.46	4.81 ± 1.18	0.015
4	Firing frequency at 200 pA, f_{200} (Hz)	4.64 ± 1.07	6.96 ± 1.53	0.023	5.87 ± 0.77	10.79 ± 1.89	0.003
5	Firing frequency at 250 pA, f_{250} (Hz)	8.57 ± 1.62	13.39 ± 2.05	0.0045	10.69 ± 0.95	17.78 ± 2.47	0.0016
6	AP threshold, V_{th} (mV)	–38.49 ±1.48	–40.8 ± 1.39	0.009	–38.9 ± 0.6	–42.9 ± 1.4	5.4×10^{-4}
7	AP peak, V_{AP}^{peak} (mV)	46.41 ± 1.84	41.05 ± 3.65	0.16	51.0 ± 1.7	43.2 ± 1.7	2.2×10^{-5}
8	AP amplitude, V_{AP} (mV)	127.72 ± 1.99	122.31 ± 4.39	0.20	126.2 ± 1.8	118.4 ± 1.9	2.0×10^{-5}
9	AP half-width, T_{APHW} (ms)	0.86 ± 0.03	0.87 ± 0.026	0.632	0.85 ± 0.02	0.82 ± 0.02	0.17
10	Peak dV/dt, $\left.\frac{dV}{dt}\right\|_{AP}^{max}$ (V/s)	467.56 ± 25.1	414.2 ± 35.6	0.23	528.5 ± 22.7	443.5 ± 20.8	9.6×10^{-4}
11	Min dV/dt, $\left.\frac{dV}{dt}\right\|_{AP}^{min}$ (V/s)	–128.2 ± 3.9	–124 ± 4.9	0.45	–125.2 ± 3.3	–128.3 ± 3.6	0.31
12	Latency to first spike, T_{1AP} (ms)	72.9 ± 6.7	56.15 ± 4.4	0.01	54.7 ± 5.6	49.2 ± 10.7	0.44
13	First ISI, T_{1ISI} (ms)	73.1 ± 6.7	64.7 ± 22	0.54	38.1 ± 9	8.5 ± 1.62	5.0×10^{-3}

	**Measurement (units)**	***p* values from two-way mixed mode ANOVA**		
		**Within cell factor (0 min *vs.* 45 min)**	**Between cells factor (Control *vs.* TBF)**	**Interaction**
1	Firing frequency at 50 pA, f_{50} (Hz)	—	—	—
2	Firing frequency at 100 pA, f_{100} (Hz)	0.260	0.249	0.260
3	Firing frequency at 150 pA, f_{150} (Hz)	0.118	0.112	0.250
4	Firing frequency at 200 pA, f_{200} (Hz)	0.015	0.295	0.364
5	Firing frequency at 250 pA, f_{250} (Hz)	0.004	0.30	0.554
6	AP threshold, V_{th} (mV)	0.0013	0.492	0.351
7	AP peak, V_{AP}^{peak} (mV)	2.8×10^{-4}	0.269	0.494
8	AP amplitude, V_{AP} (mV)	0.782	0.421	0.263
9	AP half-width, T_{APHW} (ms)	0.782	0.665	0.2627
10	Peak dV/dt, $\left.\frac{dV}{dt}\right\|_{AP}^{max}$ (V/s)	0.0047	0.209	0.489
11	Min dV/dt, $\left.\frac{dV}{dt}\right\|_{AP}^{min}$ (V/s)	0.947	0.952	0.288
12	Latency to first spike, T_{1AP} (ms)	0.088	0.375	0.378
13	First ISI, T_{1ISI} (ms)	0.0456	0.0042	0.254
